# Pathophysiology‐Directed Engineering of a Combination Nanoanalgesic for Neuropathic Pain

**DOI:** 10.1002/advs.202405483

**Published:** 2024-12-24

**Authors:** Wenkai Wang, Yan Wang, Xinle Huang, Peng Wu, Lanlan Li, Yang Zhang, Yihui Chen, Zhiyu Chen, Changqing Li, Yue Zhou, Jianxiang Zhang

**Affiliations:** ^1^ Department of Orthopedics Xinqiao Hospital Third Military Medical University (Army Medical University) Chongqing 400037 P. R. China; ^2^ Department of Orthopedics General Hospital of PLA Xizang Military Area Command Lhasa 850007 P. R. China; ^3^ Department of Pharmaceutics College of Pharmacy Third Military Medical University (Army Medical University) Chongqing 400038 P. R. China; ^4^ War Trauma Medical Center State key Laboratory of Trauma Burns and Combined injury Army Medical Center Daping Hospital Third Military Medical University (Army Medical University) Chongqing 400038 P. R. China; ^5^ Department of Orthopedics The Second Naval Hospital of Southern Theater Command Sanya 572000 P. R. China; ^6^ School of Pharmacy Hanzhong Vocational and Technical College Hanzhong 723002 P. R. China; ^7^ Department of General Surgery Xinqiao Hospital Third Military Medical University (Army Medical University) Chongqing 400037 P. R. China; ^8^ Department of Orthopedics The First Affiliated Hospital Chongqing Medical University Chongqing 400016 P. R. China; ^9^ State Key Laboratory of Trauma and Chemical Poisoning Third Military Medical University (Army Medical University) Chongqing 400038 P. R. China; ^10^ Yu‐Yue Pathology Scientific Research Center 313 Gaoteng Avenue, Jiulongpo District Chongqing 400039 P. R. China

**Keywords:** nanoanalgesic, nerve regeneration, neuroinflammation, neuropathic pain, targeted therapy

## Abstract

Neuropathic pain, one of the most refractory pain diseases, remains a formidable medical challenge. There is still an unmet demand for effective and safe therapies to address this condition. Herein, a rat model of nerve injury‐induced neuropathic pain is first established to explore its pathophysiological characteristics. Recognizing the role of neuroinflammation, an inflammation‐resolving amphiphilic conjugate PPT is designed and synthesized by simultaneously conjugating polyethylene glycol, phenylboronic acid pinacol ester, and Tempol onto a cyclic scaffold. PPT can self‐assemble into nanomicelles (termed PPTN). Following intravenous injection, PPTN preferentially accumulates in the injured nerve, ameliorates the neuroinflammatory milieu, and promotes nerve regeneration, thereby shortening neuropathic pain duration in rats. Moreover, the Ca^2+^ channel α2δ1 subunit is identified as a therapeutic target by RNA‐sequencing analysis of the injured nerve. Based on this target, a mimicking peptide (AD peptide) is screened as an analgesic. By packaging AD peptide into PPTN, a combination nano‐analgesic APTN is developed. Besides potentiated anti‐hyperalgesic effects due to site‐specific delivery and on‐demand release of AD peptide at target sites, APTN simultaneously inhibits neuroinflammation and promotes nerve regeneration by reprogramming macrophages via regulating MAPK/NF‐kB signaling pathways and NLRP3 inflammasome activation, thus affording synergistic efficacies in treating nerve injury‐induced neuropathic pain.

## Graphical abstract

A nanoanalgesic combining anti‐neuroinflammatory and pain relief properties is engineered by encapsulating an analgesic peptide into inflammation‐resolving and responsive nanomicelles. This nanotherapy demonstrates synergistic anti‐hyperalgesic and nerve regeneration effects in rats with injury‐induced neuropathic pain, mechanistically by site‐specifically blocking the Ca^2+^ channel α2δ1 and reprogramming macrophages via regulating MAPK/NF‐κB signaling. This combination of nanoanalgesic is promising for treating neuroinflammation‐related pain diseases.

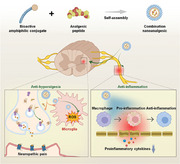

## 1 Introduction

Pain remains a pervasive medical issue worldwide, posing significant challenges for effective management.^[^ ^1^ ^]^ Currently, ≈10% of patients are diagnosed with chronic pain. Within this population, 15–25% of cases are attributed to neuropathic pain, which is closely associated with peripheral nerve injuries.^[^ ^1a^ ^]^ Neuropathic pain, resulting from diseases or lesions within the somatosensory nervous system, typically manifests following peripheral nerve injury during a trauma or surgery.^[^ ^2^ ^]^ Patients suffering neuropathic pain generally experience burning, shooting, pins, and needles in a specific skin area, persisting for more than 6 months.^[^ ^2^, ^3^ ^]^ Allodynia, hyperalgesia, and aftersensations to mechanical and thermal stimuli are typical clinical symptoms of nerve injury‐induced neuropathic pain. These symptoms frequently evolve into chronic pain conditions that severely impair patients’ quality of life. The development of neuropathic pain following nerve injury is influenced by several pathophysiological processes, including alterations in second‐order nociceptive neurons, ion channel dysfunctions, and inhibitory modulation changes, all contributing to ectopic impulses, and ultimately leading to central sensitization and neuropathic pain.^[^ ^1^, ^4^ ^]^ The current first‐line treatments for neuropathic pain encompass antidepressants (e.g., nortriptyline and amitriptyline) and antiepileptic drugs (notably gabapentin and pregabalin).^[^ ^3^, ^4^, ^5^ ^]^ However, the prolonged and excessive use of these drugs against inadequate analgesia can cause serious side effects, such as iatrogenic harm, cognitive impairment, and respiratory depression.^[^ ^6^ ^]^ Consequently, safe and effective therapies remain to be discovered and developed for neuropathic pain.

As well documented, neuroinflammation plays a critical role in mediating nerve injury‐induced neuropathic pain, by participating in the underlying pathophysiological process.^[^ ^7^ ^]^ Peripheral nerve injury mostly occurs with enclosed hemorrhage, tissue necrosis, and massive inflammatory cell infiltration, which produce extensive inflammatory mediators and trigger acute neuroinflammatory reactions. Existing evidence suggests that the accumulation of inflammatory mediators, such as histamine, bradykinin, tumor necrosis factor (TNF)‐α, substance P, and reactive oxygen species (ROS), are involved in developing neuropathic pain.^[^ ^7^, ^8^ ^]^ In addition, previous studies revealed the presence of inflammatory and immune responses in the spinal dorsal horn (SDH) following nerve injury.^[^ ^7^, ^9^ ^]^ Consequently, the elimination of the pain mediators by attenuating the neuroinflammatory reactions is an intriguing approach to pain therapy, which can remove molecules that cause sensitization.^[^ ^8b^ ^]^ Moreover, early intervention against neuroinflammation may protect spared axons and myelin sheaths from secondary injury, thereby fostering nerve repair.^[^ ^10^ ^]^ Notably, enhanced axonal repair and myelination can diminish ectopic impulses and accelerate neuropathic pain relief.^[^ ^10^, ^11^ ^]^ Based on these insights, we propose that simultaneous anti‐inflammation and inhibition of central sensitization may serve as a promising therapeutic avenue for neuropathic pain following nerve injury.

Nonsteroidal anti‐inflammatory drugs (NSAIDs) have traditionally been employed in treating inflammatory disorders. However, these agents are often linked with notable adverse effects, including cardiovascular diseases, gastrointestinal bleeding, and renal injury.^[^ ^12^ ^]^ To develop novel, safe, and effective anti‐inflammatory therapies, bioactive materials have been engineered to target and regulate specific inflammation‐correlated signaling pathways or molecules correlated with inflammation. The effectiveness of these anti‐inflammatory materials has been explored in various animal models of acute and chronic inflammation.^[^ ^13^ ^]^ Among them, specific polysaccharides and their derivatives, glycoproteins, and biomimetic peptides have garnered attention for their comprehensive anti‐inflammatory properties.^[^ ^14^ ^]^ Nonetheless, the clinical application of these biomaterial‐based anti‐inflammatory therapies faces significant hurdles due to complicated chemical structures, poor quality control, reproducible mass production challenges, insufficient potency, undetermined hydrolytic/metabolic profiles, and safety concerns. Herein, we introduce an innovative anti‐inflammatory strategy that utilizes a bioactive and amphiphilic conjugate for neuropathic pain treatment. This strategy not only offers targeted delivery to nerve injury sites but also possesses potent anti‐inflammatory and anti‐oxidative properties. Moroever, this engineered amphiphilic conjugate can self‐assemble into ROS‐responsive nanomicelles, facilitating the encapsulation and inflamamtion‐triggerable release of analgesic agents.

**Figure 1:**
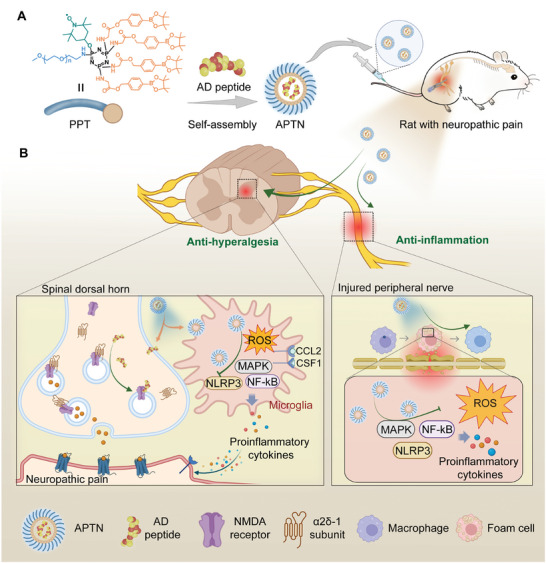
Schematic illustration of targeted treatment of neuropathic pain by a combination nanotherapy with anti‐hyperalgesic and neuroinflammation‐resolving effects. A) A sketch shows engineering of a nanotherapy with analgesic and inflammation‐resolving activities by self‐assembly of a bioactive amphiphilic conjugate (i.e., PPT) and AD peptide. B) Targeted treatment of neuropathic pain with the analgesic and anti‐inflammatory nanotherapy APTN by inhibiting ROS overproduction at the injured nerve and targeting α2δ1‐NMDA receptor complex, thus inhibiting MAPK/NF‐κB signaling pathways and reducing NLRP3 inflammasome activation. ROS, reactive oxygen species; MAPK, mitogen‐activated protein kinase; NF‐κB, nuclear factor kappa‐B; NLRP3, NOD‐like receptor thermal protein domain associated protein 3. The illustration was created with BioRender.com.

On the other hand, for pharmacological treatment of chronic neuropathic pain, NSAIDs, opioids, anti‐epileptics, and antidepressants are generally used for symptomatic management. However, these analgesics lack specific therapeutic targets and mechanisms of action.^[^ ^2^, ^4^, ^15^ ^]^ Notably, considerable adverse events often limit their use, particularly after long‐term treatment.^[^ ^6^, ^16^ ^]^ Despite a growing understanding of molecular/cellular mechanisms dominating neuropathic pain, novel therapeutic regimens capable of targeting key molecules involved in pain remain to be developed by rational design and investigation.^[^ ^17^ ^]^ Capsaicin and botulinum toxin serotype A, targeting the transient receptor potential vanilloid type‐1 (TRPV1) and calcitonin gene‐related peptide (CGRP) receptors, respectively, represent strides in this direction and have entered clinical application.^[^ ^3^, ^18^ ^]^ Yet, their efficacies are confined to certain pain conditions, such as postherpetic neuralgia and migraine. To date, the development of analgesics specifically targeting neuropathic pain caused by nerve injury remains an ongoing necessity. In this study, we established a rat neuropathic pain model by peripheral nerve injury and screened the differentially expressed genes in the dorsal root ganglion (DRG). Notably, the calcium voltage‐gated channel auxiliary α2δ1 subunit was identified as a potential analgesic target. Based on this target, a therapeutic peptide was screened for relieving neuropathilc pain. Moreover, by loading the analgesic peptide into an inflammation‐resolving micellar nanoplatform, a combination nanotherapy was engineered, which can simultaneously inhibit neuroinflammation, promote nerve regeneration, and alleviate hyperalgesia, thereby affording synergistic effects for the treatment of nerve injury‐induced neuropathic pain (**Figure** **1**).

## 2 Results

### 2.1 Establishment of a Rat Model of Neuropathic Pain Induced by Peripheral Nerve Injury

**Figure 2:**
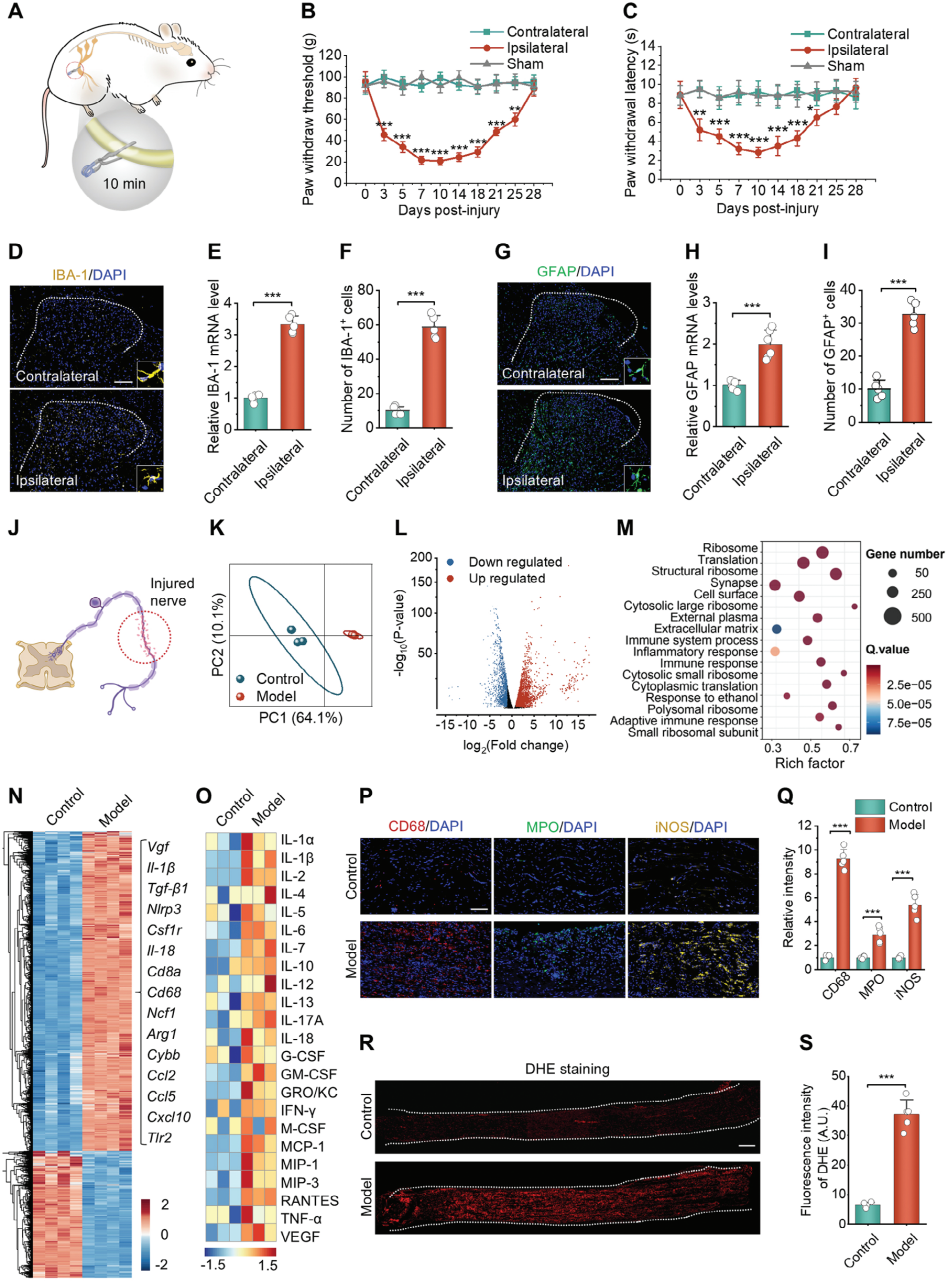
Characteristics of neuroinflammation and neuropathic pain in rats established by nerve injury. A) A schematic diagram shows the establishment of a neuropathic pain model in rats. The sciatic nerves of rats were clipped with vascular clamps for 10 min to induce nerve injury. B,C) Mechanical allodynia (B) and thermal hyperalgesia (C) emerging from days 3 to 28 after compression nerve injury, as implicated by the reduced PWT and PWL values, respectively. ^*^ *p* < 0.05, ^**^ *p* < 0.01, and ^***^ *p* < 0.001, compared to the contralateral group at the same time point. D) Immunofluorescence analysis of IBA1 in the SDH of rats at day 14 after nerve injury. Scale bar, 200 µm. E) PCR quantification of the expression of IBA1 mRNA in the spinal cords of rats at day 14 after nerve injury. F) Quantified IBA1‐positive cells in the SDH of rats at day 14 after nerve injury. G) Immunofluorescence analysis of GFAP in the SDH of rats at day 14 after nerve injury. Scale bar, 200 µm. H,I) Quantified GFAP mRNA levels (H) and GFAP‐positive cells (I) in the SDH of rats at day 14 after nerve injury. J) A sketch shows sampling of the injured nerve for RNA‐seq analysis. K) Principal component analysis (PCA) of RNA‐seq results. L) The volcano plot showing DEGs. M) GO analysis of RNA‐seq results. N) The heatmap indicates significant DEGs between the normal (control) and injured (model) nerves. O) Luminex liquid suspension chip detection of the levels of typical inflammatory mediators in the normal and injured nerves. P,Q) Immunofluorescence images indicate CD68, MPO, and iNOS (P) as well as quantification of CD68, MPO, and iNOS positive cells (Q) in the normal and injured nerves at day 3 after injury. R,S) Fluorescence images (R) and quantitative analysis (S) of DHE‐stained sciatic nerve sections. Data are expressed as means ± SD (*n* = 5). ^***^ *p* < 0.001.

Initially, we constructed a rat model of sciatic nerve injury, which was caused by temporary compression, based on the etiology of peripheral neuropathic pain observed in clinical practice, and explored the pathophysiological characteristics of this animal model (**Figure** **2**A; Figure S1A–E, Supporting Information). To validate this model, we assessed mechanical and thermal nociceptive sensitivities in rats. Mechanical hypersensitivity was quantified by the paw withdrawal threshold (PWT) using a monofilament to apply pressure to the hind paws of rats.^[^ ^19^ ^]^ A decrease in PWT indicated mechanical allodynia. Thermal sensitivity was evaluated by measuring the paw withdrawal latency (PWL), which was the time taken for a withdrawal response following thermal stimulation of the hind paws.^[^ ^20^ ^]^ A reduction in PWL was indicative of thermal hyperalgesia. After nerve injury, the PWT and PWL values on the affected side were significantly diminished. Particularly, the evolution of PWT and PWL over a 30‐day period post‐injury confirmed the successful establishment of the neuropathic pain model in rats (Figure 2B,C). Neuropathic pain syndrome appeared on day 3 post‐injury, peaked between days 7 and 14, began to subside by day 14, and resolved by day 28. In parallel, we found significantly increased microglia (Figure 2D–F) and astrocytes (Figure 2G–I) within the ipsilateral SDH after nerve injury, as evidenced by qPCR and immunofluorescence analyses. These findings implied that peripheral nerve injury caused central nociceptive sensitization within the SDH.

To elucidate the mechanisms driving pathological alterations, we conducted RNA‐sequencing (RNA‐seq) analysis on both the injured and contralateral unaffected nerves at day 7 post‐injury (Figure 2J–L). We identified 2858 significantly differentially expressed genes (DEGs) between the injured nerves and normal nerves, including 2047 upregulated (71.6%) and 811 downregulated genes (28.4%). Gene Ontology (GO) analysis revealed marked upregulation of genes associated with inflammatory and immune responses in the injured nerves (Figure 2M), encompassing pro‐inflammatory mediators (e.g., *Il‐1β*, *Il‐6*, *Il‐18*, *Nlrp3*), genes related to inflammatory cells (e.g., *Cd8a*, *Csf1r*, *Cd68*, *Arg1*), and chemoattractants (e.g., *Ccl2*, *Ccl5*, *Cxcl10*) (Figure 2N).

Following the RNA‐seq analysis, we proceeded with qPCR and ELISA to verify the inflammatory response in the injured nerve. The results showed significant upregulation of genes linked to inflammatory responses (*Il‐1β, Il‐6, Tnf‐α, Nlrp3, Tgf‐β, Csf1r, Cybb, Nox4, Arg1, Nos2, and Ccl2*) in the injured nerve relative to its normal counterpart (Figure S2A, Supporting Information). Concurrently, protein levels of IL‐1β, IL‐6, and TNF‐α were markedly elevated within 7 days post‐injury (Figure S2B–D, Supporting Information). These findings suggest that nerve injury causes acute inflammation and immune activation. In line with this, Luminex liquid suspension chip detection revealed notably up‐regulated expressions of typical proinflammatory cytokines (IL‐1α, IL‐1β, IL‐6, IL‐18, IFN‐γ, and TNF‐α) and chemokines (MCP‐1, MIP‐1, G‐CSF, GM‐CSF, and RANTES) in the injured nerve when compared to the unaffected contralateral nerve (Figure 2O). Immunofluorescence assays further revealed considerable infiltration of macrophages and neutrophils in the site of injury (Figure 2P,Q). Additionally, quantification of hydrogen peroxide (H_2_O_2_) levels and fluorescence observation of dihydroethidium (DHE)‐stained cryosections revealed elevated ROS in the injured nerve (Figure 2R,S; Figure S2E, Supporting Information). Collectively, these results demonstrated that peripheral nerve injury not only triggers neuropathic pain but also leads to acute neuroinflammation and oxidative stress in the injured nerve.

### 2.2 Engineering of an Inflammation‐Resolving and Nerve Injury‐Targeting Nanotherapy

**Figure 3:**
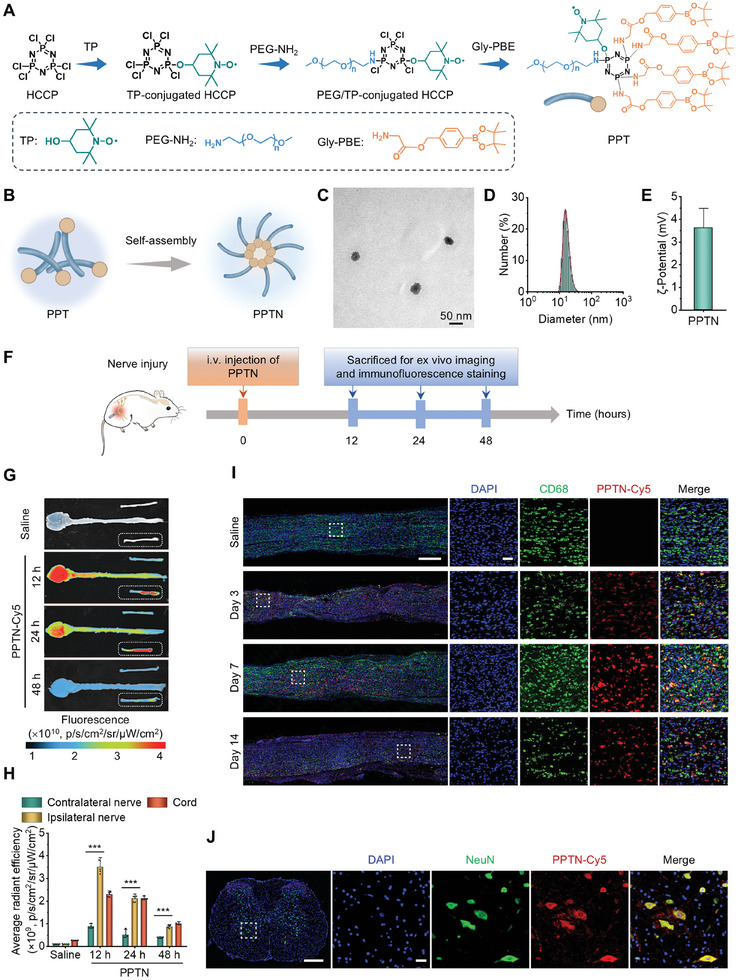
Engineering of an inflammation‐resolving nanotherapy PPTN and its targeting capability. A) Schematic illustration of the molecular structure and synthesis procedures of an inflammation‐resolving and amphiphilic conjugate (PPT). B) A sketch shows engineering of a nanotherapy (PPTN) self‐assembled by PPT. C–E) The TEM image (C), size distribution (D), and ζ‐potential (E) of PPTN. F) A workflow illustrates treatment regimens for the targeting study. G,H) Ex vivo fluorescence images (G) and quantitative data (H) show the accumulation of PPTN‐Cy5 in the injured sciatic nerve and CNS at the indicated time points after i.v. injection at day 3 after injury. I) Immunofluorescence images indicate co‐localization of PPTN‐Cy5 with CD68^+^ macrophages in longitudinal sections of sciatic nerves at different time points after injury. The right panel images show magnified regions in the white squares. All tissues were separated at 6 h after i.v. injection. Scale bars, 200 µm (left) and 20 µm (right). J) Immunofluorescence images show co‐localization of PPTN‐Cy5 with neurons in spinal cord cross‐sections at day 3 after injury. Images in the right panels illustrate the magnified region in the white square. Tissues were isolated at 6 h after i.v. injection. Scale bars, 200 µm (left) and 50 µm (right). Data in (E and H) are expressed as means ± SD (*n* = 5). ^***^ *p* < 0.001.

Considering the link between neuropathic pain and neuroinflammation, we hypothesize that facilitating inflammation resolution alongside repairing the damaged nerve can effectively mitigate neuropathic pain resulting from peripheral nerve injury. To validate this hypothesis, we initially engineered an inflammation‐resolving amphiphilic conjugate (termed PPT). This conjugate comprises polyethylene glycol (PEG), phenylboronic acid pinacol ester (PBE), and Tempol (TP), covalently conjugated onto a cyclic scaffold, forming PPT (**Figure** **3**A). Notably, PBE serves as a ROS‐responsive unit capable of eliminating H_2_O_2_. Moreover, ROS‐triggered hydrolysis of PBE can produce p‐(hydroxymethyl) phenol (HMP), a pharmacologically active compound with potent anti‐inflammatory and anti‐apoptotic properties.^[^ ^21^ ^]^ TP, as a superoxide dismutase (SOD) mimetic, can efficiently scavenge radicals and superoxide anions.^[^ ^22^ ^]^ Furthermore, PEG endows the conjugate with hydrophilicity.^[^ ^23^ ^]^

PPT was synthesized by sequential nucleophilic substitution reactions (Figure 3A; Figure S3A,B, Supporting Information). The successful synthesis of PPT was confirmed by ^1^H NMR, Fourier transform infrared (FT‐IR), and matrix‐assisted laser desorption/ionization time‐of‐flight (MALDI‐TOF) mass spectrometry (Figure S4A–C, Supporting Information). Calculation according to the ^1^H NMR spectrum revealed approximately one PEG chain, four PBE units, and one TP moiety per PPT conjugate. Due to its amphiphilic nature, PPT can be readily dissolved in aqueous solutions and spontaneously form nanomicelles by self‐assembly (Figure 3B). Transmission electron microscopy (TEM) observation demonstrated the formation of spherical nanoparticles (i.e., PPTN) from PPT (Figure 3C). Dynamic light scattering measurements indicated that the mean hydrodynamic diameter of PPTN is 31 ± 2 nm (Figure 3D), with ζ‐potential of 3.6 ± 0.9 mV (Figure 3E). In addition, PPTN exhibited excellent stability in water, saline, phosphate‐buffered saline (PBS), and standard cell culture medium (Figure S5, Supporting Information).

In line with the presence of PBE and TP moieties, PPT demonstrated the ability to scavenge various ROS types, such as H_2_O_2_ and radical, in a dose‐dependent manner (Figure S6A,B, Supporting Information). When exposed to H_2_O_2_, PPT undergoes hydrolysis, yielding water‐soluble and biocompatible components, including PEG, TP, HMP, glycine, and PO_4_^3–^. In particular, this hydrolysis process allows for the gradual release of HMP and TP (Figure S7A–C, Supporting Information). Consistently, PPT effectively reduced intracellular ROS generation in PMA‐stimulated macrophages in a dose‐response pattern (Figure S8A–D, Supporting Information). Also, PPT treatment significantly suppressed the levels of key pro‐inflammatory cytokines,^[^ ^7a^ ^]^ i.e., interleukin (IL)‐1β and IL‐6, in PMA‐induced macrophages (Figure S8E,F, Supporting Information). Collectively, these findings affirmed that PPTN exerts desirable antioxidative and anti‐inflammatory activities.

### 2.3 In Vivo Targeting Capacity of PPTN in Rats with Nerve Injury‐Induced Pain

To ascertain the in vivo targeting ability of PPTN, a rat model of neuropathic pain based on peripheral nerve injury was developed as previously described. Cy5‐labeled PPTN (PPTN‐Cy5) was prepared by co‐assembly of PPT and a Cy5‐labeled PPT conjugate, which showed a mean diameter of 43 ± 2 nm and ζ‐potential of 3.7 ± 0.8 mV (Figures S9 and S10A,B, Supporting Information). PPTN‐Cy5 was administered on day 3 after nerve injury by intravenous (i.v.) injection (Figure 3F). The peripheral nerves, brains, and spinal cords of the rats were collected at 12, 24, and 48 h post‐administration. Ex vivo imaging revealed remarkable fluorescent signals in these tissues at 12 h after injection, which remained detectable for at least 48 h (Figure 3G). Quantitative analyses indicated a significantly higher accumulation of Cy5 fluorescence within the injured nerve relative to the contralateral nerve (Figure 3H). These findings confirmed that i.v. administered PPTN preferentially accumulates in injured nerves.

Furthermore, cellular distribution profiles of PPTN‐Cy5 were examined by immunofluorescence analysis. Fluorescence observation of cryosections indicated a large number of Cy5 fluorescence‐positive cells in the injured nerves at days 3, 7, and 14 post‐injury. Moreover, we detected a pronounced co‐localization of Cy5 fluorescence with CD68‐positive macrophages in the peripheral nerves (Figure 3I; Figure S11, Supporting Information). PPTN‐Cy5 was also significantly distributed in neurons in the spinal cord (Figure 3J). Corroborating these findings, in vitro studies showed the rapid and efficient uptake of PPTN‐Cy5 by PC12 neuron‐like cells, RSC96 Schwann cells, and RAW264.7 macrophages, following dose‐ and time‐dependent patterns (Figure S10C–H, Supporting Information).

Altogether, intravenously administered PPTN can efficiently accumulate in injured nerves and the central nervous system (CNS) of rats experiencing neuropathic pain. Several mechanisms are responsible for the targeting properties of PPTN. First, neuroinflammation may trigger endothelial dysfunction and enhance vascular permeability,^[^ ^24^ ^]^ facilitating PPTN penetration through compromised blood vessels and the impaired blood‐spinal cord barrier (BSCB). Furthermore, the deteriorated integrity of the BSCB in rats with nerve injury could be partially responsible. Axonal damage has been linked to neurodegenerative variation and the subsequent disruption of the blood‐brain barrier (BBB).^[^ ^25^ ^]^ In addition, the diminutive size of PPTN is a critical factor in its accumulation within the injured neural tissue. Generally, nanoparticles under 100 nm exhibit relatively high transport across the BSCB.^[^ ^26^ ^]^ Moreover, the slight positive charge of PPTN facilitates its translocation across the negatively charged BSCB.^[^ ^25a^ ^]^ Consequently, PPTN can serve as a promising therapy or delivery vehicle to alleviate neuroinflammation and neuropathic pain, attributed to its desirable targeting capability to the site of nerve injury and SDH.

### 2.4 PPTN Attenuates Neuroinflammation and Promotes Regeneration of the Injured Nerve in Neuropathic Pain Rats

**Figure 4:**
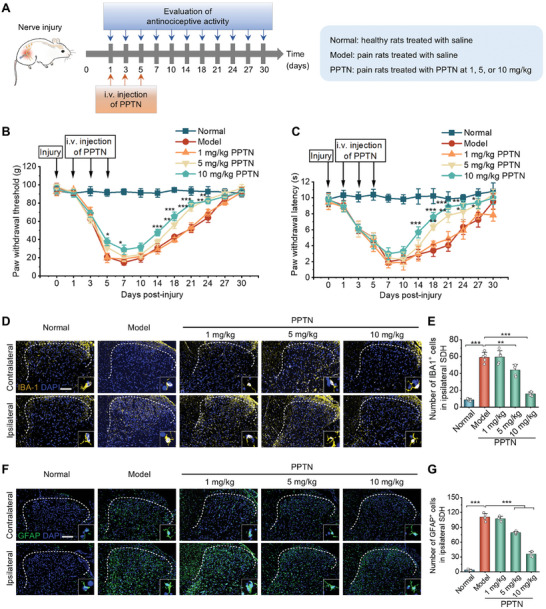
Pain‐relief effects of PPTN in rats with neuropathic pain. A) Schematic illustration of the treatment regimens. B,C) PPTN treatment alleviated mechanical allodynia (B) and heat hyperalgesia (C) induced by peripheral nerve injury. ^*^ *p* < 0.05, ^**^ *p* < 0.01, and ^***^ *p* < 0.001, compared to the model group at the same time point. D,E) Immunofluorescence images (D) and quantitative data (E) show IBA1^+^ microglia in the SDH of rats after different treatments. Scale bar, 200 µm. F,G) Immunofluorescence images (F) and quantitative data (G) indicate GFAP^+^ astrocytes in the SDH of rats after different treatments. Scale bar, 200 µm. Data are expressed as means ± SD (*n* = 5). ^**^ *p* < 0.01, ^***^ *p* < 0.001.

Based on the promising nerve injury‐targeting results, we then examined whether PPTN can ameliorate the neuroinflammatory environment and relieve neuropathic pain in rats with nerve injury (**Figure** **4**A). Behavioral tests indicated that PPTN effectively reduced mechanical and thermal hypersensitivity of pain in rats since day 14 post‐injury (Figure 4B,C). In particular, PPTN at 5 and 10 mg kg^−1^ afforded desirable outcomes. Consistently, immunofluorescence analyses of spinal cord cryosections implied that PPTN notably reduced IBA1‐positive microglia and GFAP‐positive astrocytes in the SDH (Figure 4D–G). These results suggest that PPTN may efficaciously attenuate neuroinflammation in the ipsilateral SDH of rats subjected to nerve injury.

**Figure 5:**
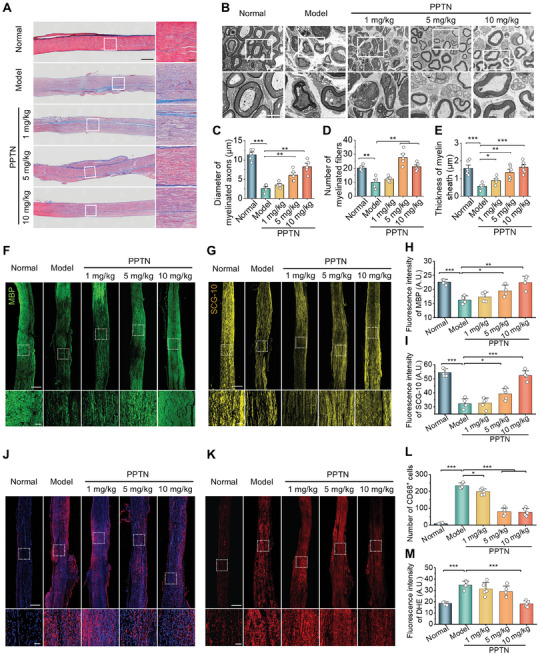
PPTN promotes neural regeneration in rats with neuropathic pain. A) Masson‐stained histological sections of sciatic nerves isolated from rats subjected to different treatments. Scale bars, 2 mm (left) and 200 µm (right). B) TEM observation of the cross‐sections of sciatic nerves after different treatments. Scale bars, 5 µm (upper) and 2 µm (lower). (C‐E) Quantification of the mean diameter of myelinated axons C), intensity of myelinated axons D), and thickness of myelin sheath E) based on TEM observation of sciatic nerves. F–I) Immunofluorescence images show the expression of MBP (F) and SCG‐10 (G) in sciatic nerves of rats after different treatments and the related quantitative data (H‐I). J–M) Immunofluorescence images (J) and micrographs of DHE‐stained cryosections (K) as well as quantitative analysis of CD68‐positive cells (L) and ROS (M) levels in sciatic nerves of rats in different groups. Scale bars in (F, G, J, and K), 2 mm (upper) and 200 µm (lower). Data are expressed as means ± SD (*n* = 5). ^*^ *p* < 0.05, ^**^ *p* < 0.01, ^***^ *p* < 0.001.

Considering that the integrity of nerve fibers is closely correlated to the development and progression of neuropathic pain, we further explored whether PPTN treatment can promote myelin sheath and axon regeneration after peripheral nerve injury. Inspection of the longitudinal sections stained with Masson showed that PPTN effectively inhibited collagen deposition and improved nerve fiber organization (**Figure** **5**A). TEM observation indicated that the diameter, thickness, and number of myelin sheaths were significantly increased after PPTN treatment, especially at 10 mg kg^−1^ (Figure 5B–E). In addition, we detected the expression of myelin basic protein (MBP, a marker protein closely related to the myelination process) and superior cervical ganglia neural‐specific 10 protein (SCG‐10, a specific protein associated with axon and dendritic growth).^[^ ^27^ ^]^ Immunofluorescence analyses implied that PPTN therapy substantially enhanced the expression of MBP and SCG‐10 (Figure 5F–I), thereby facilitating the repair of nerve fibers after injury. Additionally, PPTN considerably attenuated local inflammation and oxidative stress, as implicated by the notably reduced infiltration of CD68^+^ macrophages and diminished ROS generation (Figure 5J–M).

Collectively, these results confirmed that PPTN can effectively promote the regeneration of injured nerves by inhibiting local inflammatory and oxidative responses, thus diminishing the duration of neuropathic pain induced by nerve injury.

### 2.5 Screening of the Ca^2+^ Channel α2δ1 Subunit as an Analgesic Target for Neuropathic Pain

**Figure 6:**
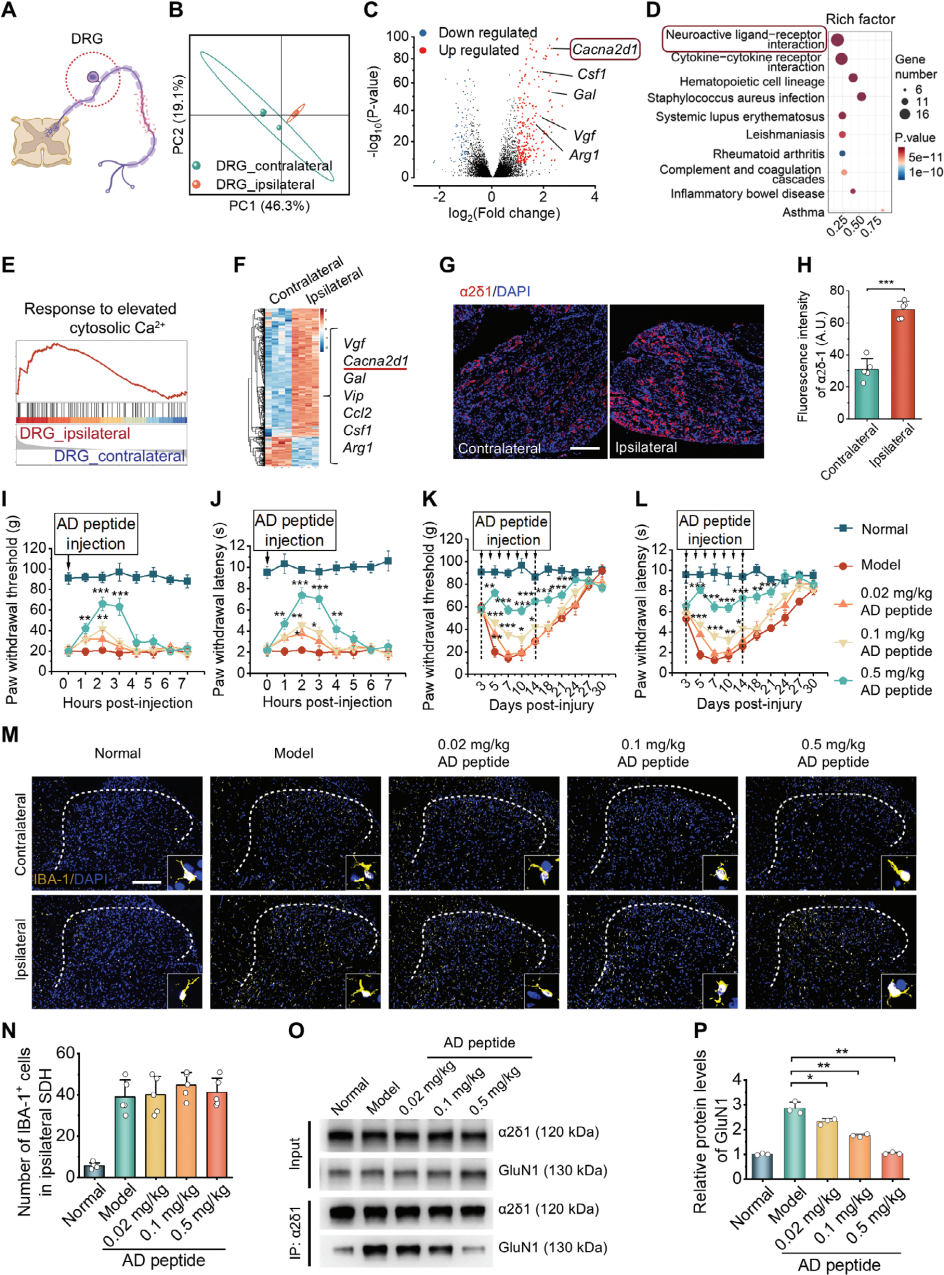
Screening of a pain‐relief AD peptide and its therapeutic effects. A) Schematic illustration of sampling the injured nerve tissue DRGs for RNA‐seq analysis. B–D) PCA analysis (B), volcano plot (C), and GO analysis (D) of RNA‐seq results. E) GSEA indicates response to elevated cytosolic Ca^2+^ for the ipsilateral and the contralateral DRGs. F) The heatmap shows significant DEGs between the ipsilateral and contralateral DRGs. G,H) Representative immunofluorescence images (G) and quantitative data (H) indicating α2δ1 levels in the DRGs of rats at day 14 after nerve injury. Scale bar, 200 µm. I–L) AD peptide treatment alleviated mechanical allodynia (I,K) and thermal hyperalgesia (J,L) during short‐term (I–J) and long‐term (K–L) examination in rats with neuropathic pain induced by nerve injury. ^*^ *p* < 0.05, ^**^ *p* < 0.01, and ^***^ *p* < 0.001, compared to the model group at the same time point. M,N) Immunofluorescence images (M) and quantitative data (N) show IBA1^+^ microglia in the SDH of rats after different treatments. Scale bar, 200 µm. O,P) Co‐immunoprecipitation analysis (O) and quantitative data (P) show the reduced expression of α2δ1‐bound NMDARs in the spinal cord of rats after nerve injury and treatment with AD peptide. Data are expressed as means ± SD (H–L and N, n = 5; P, n = 3). ^*^ *p* < 0.05, ^**^ *p* < 0.01, ^***^ *p* < 0.001.

Subsequently, we sought to identify analgesic targets and screen therapies for neuropathic pain. Persistent neuropathic pain generated by nerve injury is closely associated with the maladaptive plasticity of sensory neurons. A number of molecular mediators can induce the sensitization of nociceptive pathways.^[^ ^3^, ^4^ ^]^ In order to explore and identify therapeutic targets linked to nerve injury‐induced neuropathic pain, RNA‐seq analysis was performed for the DRGs, where spinal cord sensory afferent neurons are located (**Figure** **6**A). We identified 332 DEGs in the ipsilateral DRGs relative to the contralateral tissue (Figure 6B,C). These DEGs included neuropeptides (e.g., *Gal*, *Vip*, *Vgf*, *Npy*), pain development receptors (e.g., *Gabar5*, *Npy2r*, *Bdkrb2*), and glial cell‐related cytokines (e.g., *Ccl2*, *Il6*, *Csf1*, *Arg1*), agreeing with the activation of microglia and astrocytes in SDH. Also, GO analysis revealed that the term neuroactive ligand‐receptor interactions was highly enriched in the ipsilateral DRGs (Figure 6D), implying the development of central sensitization in the spinal cord.^[^ ^4^ ^]^ Interestingly, gene set enrichment analysis (GSEA) showed an upregulation of the gene set of responses to elevated cytosolic Ca^2+^ within the ipsilateral DRGs (Figure 6E). Previous studies demonstrated that voltage‐gated Ca^2+^ channels intensively shape cellular neurotransmission in the somatosensory nociception.^[^ ^28^ ^]^ Of note, *Cacna2d1*, i.e., the coding gene of the voltage‐gated Ca^2+^ channel α2δ1 subunit (abbreviated as α2δ1), was significantly upregulated in the ipsilateral DRGs (Figure 6F–H). As a protein closely associated with nociception, α2δ1 is also a target of gabapentinoid analgesics, such as gabapentin and pregabalin. Moreover, our previous results demonstrated that gabapentin treatment significantly suppressed α2δ1 expression, concomitant with notable analgesic effects.^[^ ^29^ ^]^ Collectively, we identified α2δ1 as a potential therapeutic target. Nonetheless, severe adverse reactions, including iatrogenic harm, cognitive impairment, and respiratory depression, have been reported with gabapentinoids.^[^ ^6a,b^ ^]^ Accordingly, site‐specific delivery of gabapentinoids is preferred to minimize their side effects.

Recently, increasing evidence has substantiated that α2δ1 mediates neuropathic pain by interacting with NMDA receptors,^[^ ^30^ ^]^ which can be uncoupled by a specific mimicking peptide (VSGLNPSLWSIFGLQFILLWLVSGSRHYLW, termed as AD peptide), thereby resulting in analgesia.^[^ ^30^, ^31^ ^]^ Therefore, we evaluated the therapeutic effects of AD peptide in rats with nerve injury‐induced neuropathic pain. The analgesic effects of AD peptide were assessed by detecting the acute variations and long‐term baseline of PWT and PWL in pain rats. Of note, the acute analgesic effect was examined by measuring the PWT and PWL values after a single injection of different formulations. Changes in PWT and PWL within the whole course of treatment suggest the long‐term effects of AD peptide. Considering that AD peptide is a highly hydrophobic peptide with poor solubility in aqueous solutions, it was administered by intraperitoneal (i.p.) injection to minimize the side effects associated with repeated i.v. administration. After a single i.p. injection in pain rats, AD peptide at 0.1 or 0.5 mg kg^−1^ significantly reduced PWT and PWL of the ipsilateral hind paw caused by nerve injury (Figure 6I,J). Consistently, daily i.p. injections of AD peptide at 0.1 or 0.5 mg kg^−1^ in pain rats generated a gradual increment in the baseline of PWT and PWL (Figure 6K,L), suggesting that AD peptide can inhibit mechanical allodynia and thermal hyperalgesia. These results demonstrate the desirable analgesic activity of AD peptide. Further, to elucidate the therapeutic mechanisms of AD peptide, we examined the activation of microglia in the SDH and assessed the α2δ1‐NMDAR interaction. Immunofluorescence analysis of spinal cord cryosections revealed that treatment with AD peptide did not attenuate microglial activation, which mainly contributes to neuroinflammation in the SDH (Figure 6M,N). By contrast, co‐immunoprecipitation showed that AD peptide inhibited the α2δ1‐NMDAR interaction in a dose‐dependent manner (Figure 6O,P). Consequently, these findings suggested that AD peptide alleviates neuropathic pain by disrupting the α2δ1‐NMDAR interaction rather than by regulating the neuroinflammatory environment.

### 2.6 Engineering of an Analgesic and Inflammation‐Resolving Nanotherapy Targeting α2δ1

**Figure 7:**
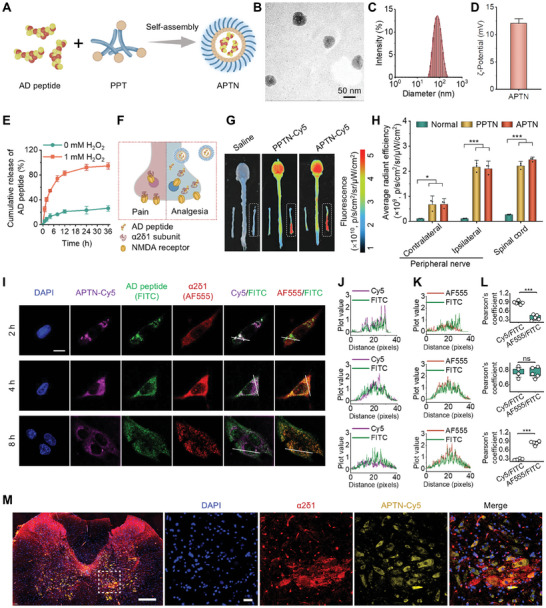
Engineering, characterization, and targeting capability of a pain‐relieving and inflammation‐resolving nanotherapy APTN. A) Schematic depicting the design and engineering of APTN based on the inflammation‐resolving conjugate PPT and AD peptide. B–E) Typical TEM image (B), size distribution (C), ζ‐potential (D), and in vitro release profiles (E) of APTN. F) Schematic illustration of APTN inhibiting pain hypersensitivity via disrupting the α2δ1‐NMDAR complex. G, H) Ex vivo fluorescence images (G) and quantitative data (H) showing the accumulation of APTN‐Cy5 in the injured sciatic nerves and CNS after i.v. injection at day 3 after injury. I) Confocal microscopic images indicate cellular uptake of APTN‐Cy5 and triggerable release of FITC‐labeled AD peptide in PC12 neuronal cells. α2δ1 was labeled with Alexa Fluor 555 (AF555). Scale bar, 5 µm. J,K) Fluorescence intensity profiles of FITC compared with Cy5 (J) and AF555 (K) in the radial direction of the white lines at different time points. L) Pearson's correlation analysis of the co‐localization coefficient of FITC with Cy5 or AF555. M) Representative immunofluorescence images show co‐localization of APTN‐Cy5 with α2δ1^+^ neurons in cross‐sections of the spinal cord at day 3 after injury. Regions in the white squares are magnified and illustrated in the right panels. In this case, spinal cords were isolated at 6 h after i.v. injection. Scale bars, 200 µm (left) and 20 µm (right). Data are expressed as means ± SD (D, E, and H, n = 3, J‐L, n = 5). ^*^ *p* < 0.05, ^***^ *p* < 0.001; ns, no significance.

As a hydrophobic peptide, AD peptide exhibits limited solubility in aqueous solution and a short plasma half‐life in blood circulation. Moreover, the AD peptide itself cannot efficiently penetrate through the BBB and has no nerve‐injury targeting capability. Based on the above findings on nerve injury‐targeting and inflammation‐resolving effects of PPTN, we reasonably speculate that PPTN can serve as a versatile nanovehicle to realize site‐specific delivery and ROS‐triggerable release of AD peptide as well as afford synergistic neuroinflammation resolution, nerve injury repair, and pain‐relief effects. As a proof of concept, AD peptide‐loaded PPTN (defined as APTN) was prepared by self‐assembly (**Figure** **7**A). To optimize the preparation conditions for APTN, we primarily screened the weight ratio of AD peptide to PPT. By fabricating APTN nanomicelles based on various PPT/AD peptide weight ratios, we assessed the AD peptide encapsulation efficiency and drug loading capacity. The results revealed that the optimal encapsulation rate and drug loading capacity were achieved at a PPT/AD peptide weight ratio of 25:1 (Table S1, Supporting Information). For APTN derived from the optimized formulation, TEM observation revealed a spherical morphology (Figure 7B), with a relatively narrow size distribution (Figure 7C). The mean diameter of APTN was 72 ± 1 nm, while its ζ‐potential was 11.9 ± 0.3 mV (Figure 7D). The loading content of AD peptide in APTN was 33.2 µg mg^−1^. These results suggested that PPTN can efficiently package AD peptide, mainly by hydrophobic interactions. Further, in vitro tests indicated slow release of AD peptide from APTN in PBS without H_2_O_2_ (Figure 7E). By contrast, dramatically rapid release occurred in PBS with 1 mM H_2_O_2_. Accordingly, PPTN enabled the ROS‐responsive release of the loaded AD peptide molecules.

Then, we examined the targeting capability of APTN. Given that the AD peptide is a therapeutic agent targeting the α2δ1‐NMDAR complex (Figure 7F),^[^ ^30a^ ^]^ we conducted both in vitro and in vivo evaluations of APTN targeting performance (Figure 7G–M). Initially, the cellular uptake and colocalization of Cy5‐labeled PPTN containing FITC‐labeled AD peptide (termed APTN‐Cy5) were observed in PC12 neuron‐like cells using confocal microscopy. α2δ1 was identified using immunofluorescence, stained with a fluorescent dye Alexa Fluor 555 (AF555) (Figure 7I). Two hours post‐incubation, fluorescence signals due to Cy5 and FITC exhibited notable co‐localization, which was gradually decreased over 4 and 8 h, indicating the release of AD peptide from PPTN (Figure 7J–L). Meanwhile, whereas FITC and AF555 fluorescence showed relatively low co‐localization at 2 h, the corresponding co‐localization coefficient considerably increased at 4 and 8 h (Figure 7J–L). This revealed notable binding of released AD peptide with α2δ1.

For in vivo targeting studies, APTN‐Cy5 or PPTN‐Cy5 was administered to rats via i.v. injection on day 7 after peripheral nerve injury (Figure 7G). Six hours post‐administration, remarkable fluorescence signals were observed in the CNS and injured nerve tissues of rats treated with APTN‐Cy5 and PPTN‐Cy5. Nevertheless, quantitative analyses indicated no significant differences in the fluorescence intensity within the examined tissues between the two groups (Figure 7H). Moreover, immunofluorescence staining of cryosections of spinal cord tissues showed that the majority of Cy5‐labeled cells were also positive for α2δ1 (Figure 7M). Also, we compared the accumulation efficiency of APTN in the CNS under physiological and neuropathic pain conditions. At 12 h after i.v. injection of APTN‐Cy5 in healthy rats, ex vivo imaging revealed notable fluorescence in the brain and spinal cord of healthy rats (Figure S12A, B, Supporting Information), which was significantly stronger than that of the saline‐treated control group. Confocal microscopy observation of spinal cord tissue sections also indicated the cellular distribution of APTN‐Cy5 in the spinal cord neurons (Figure S12C,D, Supporting Information). This brain accumulation capability of APTN is likely attributed to its unique physicochemical properties, particularly its small size and slight positive charge. Nevertheless, the accumulation efficiency of APTN‐Cy5 in the CNS of healthy rats was significantly lower compared to that observed in rats with peripheral nerve injury. Taken together, these results suggest that APTN can cross the BBB, particularly in the context of nerve injury, release the loaded AD peptide within neurons, and ultimately enable targeted delivery of AD peptide to α2δ1.

### 2.7 Therapeutic Effects of APTN in Rats with Nerve Injury‐Induced Neuropathic Pain

**Figure 8:**
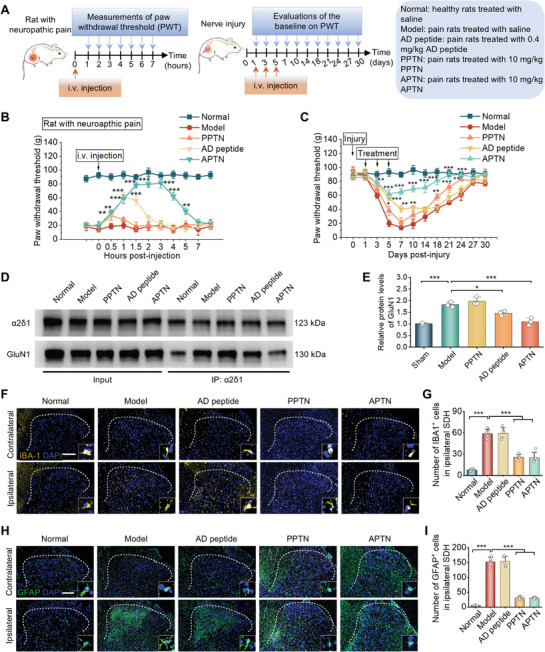
Pain‐relief effects of APTN in rats with neuropathic pain. A) Schematic illustration of the treatment regimens. The acute analgesic effect of APTN was assessed within 7 h after i.v. injection in rats with neuropathic pain (left). The long‐term analgesic effect of APTN was evaluated by testing the baseline of PWT in rats with nerve injury from days 1 to 30 (right). B) Acute anti‐hyperalgesic activities after a single i.v. injection of different formulations in rats with neuropathic pain on day 7 after nerve injury. Anti‐hyperalgesic activity is characterized by the increased PWT. C) Long‐term analgesic effects of different treatments in rats with nerve injury. ^*^ *p* < 0.05, ^**^ *p* < 0.01, and ^***^ *p* < 0.001 in (B,C), compared to the model group at the same time point. D,E) Co‐immunoprecipitation analysis (D) and quantitative data (E) showing the expression of α2δ1‐bound NMDARs in the spinal cord of rats after different treatments. F–I) Immunofluorescence images (F, H) and quantitative data (G, I) show IBA1^+^ microglia (F–G) and GFAP^+^ astrocytes (H–I) in the SDH of rats after different treatments. Scale bar, 200 µm. Data are expressed as means ± SD (B, C, G, and I, n = 5; E, n = 3). ^*^ *p* < 0.05, ^***^ *p* < 0.001.

To investigate whether encapsulation in PPTN enhances the analgesic effects of AD peptide via targeted delivery, efficacies of free AD peptide, PPTN, and APTN were compared after i.v. administration in rats with nerve injury (**Figure** **8**A). We first evaluated the acute analgesic effects of different formulations in rats with neuropathic pain after a single injection. Compared to the model group, PPTN did not significantly improve the mechanical pain thresholds, suggesting that it had no direct effects on mechanical hyperalgesia (Figure 8B). Free AD peptide at 0.4 mg kg^−1^ enhanced PWT after 1 h, followed by a decline to the baseline after 2 h. Compared to AD peptide, APTN at 0.4 mg kg^−1^ AD peptide more effectively inhibited hyperalgesia, with complete relief being maintained for 3 h. Subsequently, long‐term analgesic effects of different formulations were compared after three times of i.v. injection at days 1, 3, and 7 post‐injury. In this case, treatment with free AD peptide, PPTN, and APTN attenuated the decrease in the baseline of PWT induced by nerve injury to varied degrees (Figure 8C). This indicated that all three formulations exhibited analgesic effects. However, no significant analgesic effects were found for free AD peptide at day 14 after injury. Similar to the results mentioned above, PPTN effectively reduced pain duration, although its immediate and direct analgesic effect was lower than that of AD peptide. Comparatively, APTN showed the best efficacy in terms of both immediate analgesic activity and sustained analgesic action.

Since AD peptide exerts its pain‐relief activity by disrupting the α2δ1‐NMDAR complex, we performed coimmunoprecipitation to evaluate the interaction between NMDAR and α2δ1. Compared to the model group, both free AD peptide and APTN significantly decreased the coupled protein level of NMDAR (Figure 8D,E). Notably, APTN more effectively inhibited the formation of the α2δ1‐NMDAR complex. Meanwhile, we conducted in vitro experiments to evaluate the effects of AD peptide on the voltage‐activated calcium channel (VACC) in the neuronal cell membrane, to assess whether the disruption of the α2δ1‐NMDAR complex by AD peptide causes changes in cellular ion channels. Using confocal microscopy, we employed calcium imaging techniques to detect the impact of AD peptide on calcium influx in neurons. The results showed that both AD peptide and APTN inhibited calcium influx in depolarized neurons (Figure S13, Supporting Information). In addition, higher doses of AD peptide and APTN resulted in more pronounced inhibitory effects. These findings suggest that AD peptide can competitively bind to α2δ1, block the formation of the α2δ1‐NMDAR complex, and partially inhibit the function of neuronal VACC, thereby reducing calcium influx and exerting an analgesic effect. It is worth noting that at the same dose of AD peptide, APTN exhibited a greater inhibitory effect on calcium influx compared to free AD peptide. This enhanced inhibition can be partly attributed to the solubilizing effect of TPTN on AD peptide, which improves cellular uptake and intracellular release of more AD peptide molecules.

Additionally, immunofluorescence analyses of spinal cord cryosections indicated that both PPTN and APTN significantly reduced the number of activated microglia and astrocytes in the ipsilateral SDH of the injured nerve (Figure 8F–I). Previous studies have shown that following axonal injury, chemokines such as C‐C motif chemokine ligand 2 (CCL2) and colony‐stimulating factor 1 (CSF1) released by neurons can activate microglia, leading to neuroinflammation and the development of neuropathic pain.^[^ ^32^ ^]^ Microglia exacerbate neuroinflammation by secreting ROS and pro‐inflammatory mediators. PPTN can inhibit the neuroinflammatory response by scavenging ROS and suppressing the pro‐inflammatory polarization of microglia, thereby exerting an inhibitory effect on microglial activation. Additionally, PPTN may promote nerve injury repair and reduce the release of chemokines from neurons, which decreases the activation factors for microglia and ultimately lessens the extent of microglial activation. These results strongly demonstrated that PPTN can significantly improve the analgesic activity of AD peptide and afford remarkable synergistic effects.

**Figure 9:**
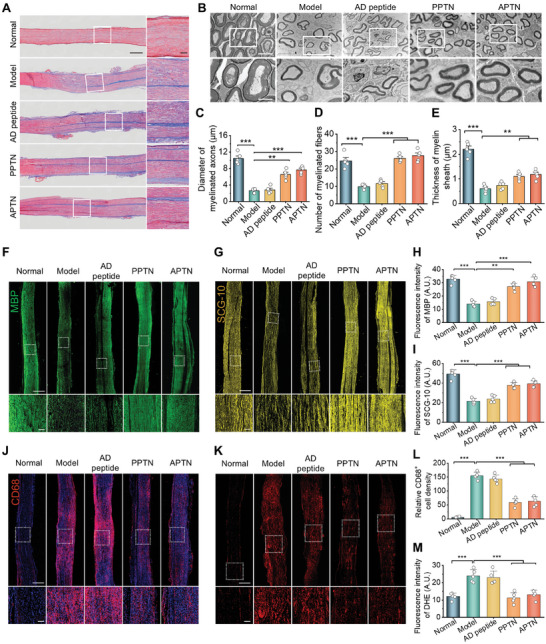
APTN promotes neural regeneration in rats with neuropathic pain. A) Masson‐stained histological sections of sciatic nerves from rats after different treatments. Scale bars, 2 mm (left) and 200 µm (right). B) TEM observation of the cross‐sections of sciatic nerves. Scale bars, 5 µm (upper) and 2 µm (lower). (C–E) Quantification of the mean diameter of myelinated axons C), intensity of myelinated axons D), and thickness of myelin sheath E) based on TEM images of sciatic nerves. F–I) Immunofluorescence images of MBP (F) and SCG‐10 (G) in sciatic nerves of rats after different treatments and the related quantitative data (H–I). J–M) Immunofluorescence images (J) and micrographs of DHE‐stained cryosections (K) as well as quantitative analysis of CD68 (L) and ROS (M) in sciatic nerves of rats in different groups. Scale bars in (F, G, J, and K), 2 mm (upper) and 200 µm (lower). Data are expressed as means ± SD (*n* = 5). ^**^ *p* < 0.01, ^***^ *p* < 0.001.

In view of the fact that the improved regeneration of injured nerve fibers can promote neuropathic pain relief, we also assessed the possible beneficial effects of APTN on nerve injury. After compression injury, the sciatic nerve structure was destroyed, and the myelin was disintegrated, showing fiber fracture and edema. Compared to AD peptide, PPTN and APTN more effectively improved the organization of nerve fiber bundles and reduced pathological collagen fibers (**Figure** **9**A). TEM observation suggested that treatment with PPTN and APTN notably promoted myelin sheath regeneration (Figure 9B–E), while comparable demyelination was found for the AD peptide and model groups. Furthermore, immunofluorescence analysis revealed considerably high levels of MBP and SCG‐10 in sciatic nerve sections of both PPTN and APTN groups (Figure 9F–I), indicating significantly activated repair of the injured myelin sheath and axon in these two groups, as compared to the free AD peptide group. Consistently, intervention with PPTN and APTN effectively attenuated inflammation and oxidative stress in the sciatic nerve, as implicated by markedly decreased macrophage infiltration and ROS generation (Figure 9J–M). In these cases, free AD peptide afforded outcomes similar to those of the model group. These results collectively substantiated that PPTN potentiated the analgesic effect of AD peptide mainly by promoting repair and regeneration of the injured nerve, resulting from its anti‐neuroinflammatory and anti‐oxidative effects.

### 2.8 Mechanistic Studies

#### 2.8.1 APTN Reverses the Pro‐Inflammatory Polarization of Macrophages After Phagocytosis of Myelin Debris

**Figure 10:**
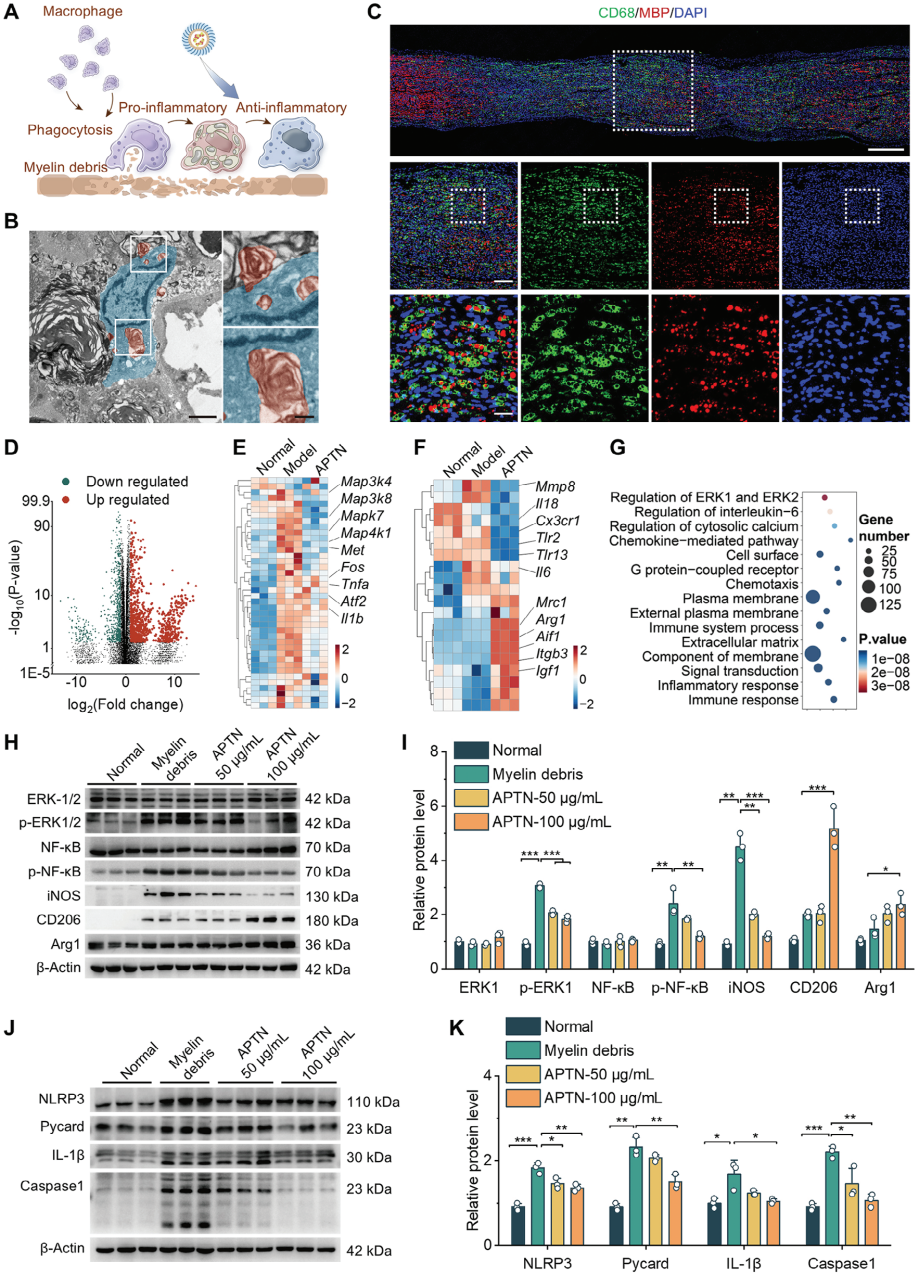
APTN reprograms macrophages after phagocytosis of myelin debris. A) Schematic illustration of APTN inhibiting polarization of pro‐inflammatory macrophages. B) TEM observation of macrophages (teal blue) and myelin debris (brown) in sciatic nerves at day 3 after injury. Regions in the white squares are magnified and illustrated in the right panels. Scale bars, 2 µm (left) and 0.2 µm (right). C) Representative immunofluorescence images show co‐localization of MBP with CD68^+^ macrophages in longitudinal sections of sciatic nerves on day 3 after injury. Regions in the white squares are magnified and illustrated in the middle and lower panels. Scale bars, 500 µm (upper), 100 µm (middle), and 20 µm (lower). D–G) The volcano plot (D), heatmap (E–F), and GO analysis (G) of RNA‐seq results of myelin debris‐phagocytosed BMDMs with or without treatment with APTN. H,I) Typical Western blotting bands (H) and quantitative analysis (I) of proteins associated with MAPK/NF‐κB signaling pathways and macrophage polarization. J,K) Western blotting analysis of biomarkers relevant to the NLRP3 inflammasome activation. Data are expressed as means ± SD (*n* = 3). ^*^ *p* < 0.05, ^**^ *p* < 0.01, ^***^ *p* < 0.001.

Macrophages are one of the major inflammatory cells involved in the repair and regeneration of the injured nerve. However, recent studies suggested that the over‐activation of macrophages may aggravate neuroinflammation and tissue damage (**Figure** **10**A).^[^ ^33^ ^]^ In addition, phagocytosis of myelin particles (i.e., myelin debris released from damaged primary afferents) by microglia in the SDH contributes to neuropathic pain after peripheral nerve injury. Accordingly, we examined whether phagocytosis of myelin particles by macrophages occurs in the injured nerve. On day 7 after compression injury, TEM observation of the cross‐section of the injured nerve clearly showed phagocytosed myelin debris in macrophages (Figure 10B). Consistently, immunofluorescence analysis revealed the co‐localization of CD68 and MBP, which further confirmed the phagocytosis of myelin debris in macrophages (Figure 10C). This suggests that macrophage phagocytosis performance is involved in the neuroinflammatory response and tissue repair after nerve injury.

Using bone marrow‐derived macrophages (BMDMs) and spinal myelin debris, we confirmed the phagocytosis of myelin debris by macrophages in vitro. Nevertheless, treatment of BMDMs with ATPN did not affect myelin debris phagocytosis performance. qPCR and Western blot analyses revealed enhanced expressions of IL‐6, TNF‐α, and inducible nitric oxide synthase (iNOS) in BMDMs with phagocytosed myelin debris relative to the control BMDMs (Figure S14A–C, Supporting Information), implicating pro‐inflammatory polarization of BMDMs. By contrast, APTN treatment significantly inhibited the pro‐inflammatory cytokine production in myelin debris‐laden BMDMs and promoted the expression of arginase 1 (Arg1) and CD206 (Figure S14D,E, Supporting Information), indicating anti‐inflammatory polarization of macrophages. Accordingly, APTN can effectively reverse myelin debris‐mediated pro‐inflammatory activation of macrophages.

#### 2.8.2 APTN Reprograms Macrophages by Inhibiting MAPK/NF‐ĸB Signaling Pathways

To further address the anti‐neuroinflammatory mechanisms of APTN, we performed RNA‐seq analysis for BMDMs containing phagocytosed myelin debris, with and without APTN treatment. We identified 660 upregulated genes and 524 downregulated genes when myelin debris‐laden BMDMs were compared with their normal counterparts (Figure 10D). Meanwhile, APTN treatment resulted in 208 genes being upregulated and 400 genes being downregulated. Following treatment with APTN, M1 phenotype‐associated genes (like *Tnf‐α*, *Il‐1β*, *Nos2*, *Nfatc3*, and *Atf2*) were significantly inhibited, while M2 phenotype‐related genes (such as *Arg1*, *Msr1*, *Aif1*, and *Igf1*) were notably increased (Figure 10E,F). These results verified the macrophage reprogramming effects of APTN. In particular, Kyoto Encyclopedia of Genes and Genomes (KEGG) analysis revealed significant enrichment of the MAPK and NF‐κB signaling pathways after APTN treatment (Figure 10G), which are intimately associated with inflammatory responses and M1 macrophage polarization.^[^ ^34^ ^]^ Additionally, NF‐κB signaling is known to activate the NLR family pyrin domain‐containing 3 (NLRP3) inflammasome,^[^ ^35^ ^]^ a key driver of inflammation. Furthermore, Western blot analysis showed that APTN significantly attenuated the phosphorylation of MAPK and NF‐κB, reduced iNOS expression, and reinforced Arg1 and CD206 expression in a dose‐dependent manner (Figure 10H,I). Correspondingly, APTN reduced the expression of IL‐1β, NLRP3, Pycard, and caspase‐1 (Figure 10J,K). These data demonstrate that APTN can reprogram macrophages by regulating the MAPK/NF‐kB signaling pathway and inhibiting activation of NLRP3.

### 2.9 Safety Studies of APTN

Finally, the safety profiles of APTN were tested. In vitro assays utilizing PC12 cells revealed minimal cytotoxicity associated with APTN, even at a high dose of 1 mg mL^−1^ (Figure S15, Supporting Information). Further, in vivo studies were conducted in healthy rats. Following a single i.v. administration of APTN at either 50 or 100 mg kg^−1^, no notable alterations in body weight were observed (Figure S16A, Supporting Information). Fourteen days post‐administration, the organ index values of the heart, lung, liver, spleen, and kidneys showed no significant changes across all groups (Figure S16B, Supporting Information). Furthermore, rats treated with APTN exhibited no significant deviations in biomarkers indicative of hepatic and renal functions when compared to those treated with saline (Figure S16C–J, Supporting Information). Additionally, histological examination of H&E‐stained sections from the major organs indicated no significant pathological alterations or injuries in any of the rats subjected to different treatments (Figure S17, Supporting Information).

## 3 Discussion

Neuropathic pain induced by nerve injury often progresses into chronic pain conditions with a severe impact on patients’ quality of life for years. Globally, neuropathic pain remains an arduous clinical problem.^[^ ^1a^ ^]^ Previous studies ignored the role of neuroinflammatory responses in the development of neuropathic pain, which largely contributes to the transition into chronic diseases. This is the reason why NSAIDs are not recommended for the treatment of neuropathic pain. Moreover, the disruption of myelin and axonal structure caused by neuroinflammation is also responsible for the pathogenesis of neuropathic pain. Impaired regeneration after the loss of myelin integrity and axonal conduction can lead to painful neuropathy.^[^ ^36^ ^]^ Therefore, eliminating neuroinflammation and accelerating myelination and axon maturation of the injured nerve represent intriguing strategies for alleviating neuropathic pain. Recently, a growing number of anti‐inflammatory therapies have been investigated to promote peripheral nerve repair and alleviate neuropathic pain. Compared to traditional anti‐inflammatory drugs (such as NSAIDs and glucocorticoids), biomaterials with intrinsic inflammation‐resolving capacity are promising for the treatment of different inflammatory diseases due to their desirable activities, ease of process into different formulations, versatility for combination with various drugs, and few side effects. According to the neuroinflammation feature of never injury‐induced neuropathic pain in rats, herein, we first designed and developed a hydrolysable and biocompatible inflammation‐resolving conjugate PPT. PPT can self‐assemble into a micellar nanotherapy PPTN. Therapeutically, PPTN can effectively target the injured nerve, inhibit neuroinflammation, promote nerve repair, and reduce pain duration in neuropathic pain rats with nerve injury. Instead of treating the symptoms merely, PPTN removed mediators that trigger nociceptors and cause sensitization, thus representing an etiology‐oriented strategy for pain relief. Of note, PPTN obviously reduced the activation of microglia in the SDH, inhibited the sensitization of sensory neurons, and effectively shortened the course of pain. To be emphasized, the anti‐inflammatory effects of PPTN are mainly attributed to its ability to scavenge ROS and inhibit the activation of pro‐inflammatory cells. By attenuating inflammation, PPTN promotes nerve repair, which in turn yields analgesic effects. Nevertheless, this therapeutic process is inherently gradual and does not provide immediate acute analgesic relief.

Currently, available first‐line analgesics for neuropathic pain mainly alleviate central sensitization. Antidepressants and serotonin‐norepinephrine reuptake inhibitors realize analgesia via inhibiting presynaptic reuptake and activating descending aminergic pathways.^[^ ^4^ ^]^ Besides, the analgesic effects of gabapentinoids and calcium channel antagonists involve the inhibition of calcium signaling and excitatory transmitter release.^[^ ^5^ ^]^ Despite their effectiveness to varied degrees, these drugs mostly possess wide‐ranging therapeutic targets, thus leading to a series of side effects and inadequate pain relief in tolerated doses.^[^ ^3^, ^6^, ^37^ ^]^ By establishing a rat model of neuropathic pain with transient compression injury, we identified a specific therapeutic target, i.e., the voltage‐gated calcium channel α2δ1 subunit, which is one of the recognized classical targets for gabapentinoids. However, these analgesics show no specific targets for the treatment of neuropathic pain.^[^ ^2^, ^3^, ^4^ ^]^ Recent studies demonstrated that the interaction between α2δ1 and NMDA receptors is mainly responsible for pain induced by α2δ1.^[^ ^30a^ ^]^ Furthermore, AD peptide, derived from the C‐terminal domain of α2δ1, can disrupt the α2δ1‐NMDAR complex to reverse pain hypersensitivity.^[^ ^30^ ^]^ Our study also indicated that AD peptide effectively alleviated hyperalgesia in rats with nerve injury‐induced neuropathic pain. To overcome the pharmaceutical limitations associated with AD peptides, such as poor water solubility, short half‐life, and systemic distribution, it was loaded into the micellar nanovehicle PPTN to achieve site‐specific delivery, ROS‐triggerable release, and synergistic anti‐hyperalgesic effects. The engineered combination nanotherapy APTN could also effectively accumulate in the injured nerve and spinal cord neurons. Compared to free AD peptide, APTN showed more effective anti‐hyperalgesic potency and a significantly prolonged duration of pain relief, mainly resulting from improved bioavailability, targeted delivery, and on‐demand peptide release. Therefore, the nanotherapy APTN enables precision analgesia for the treatment of nerve injury‐induced neuropathic pain. Notably, while there was no significant difference in the H_2_O_2_ levels between the injured side and the control side at day 5 post‐injury, this does not imply that the accumulation of ROS induced by the injury was limited to just 5 days. Our previous studies showed that a substantial number of macrophages continued to infiltrate the injured area even at day 28 post‐injury.^[^ ^29^ ^]^ This indicates that both the inflammatory response and ROS accumulation persisted locally at the site of nerve injury for up to 28 days. Additionally, following axonal injury, neurons secrete large amounts of CCL2 and CSF1, which activate surrounding microglia, a process that can continue for 30 days or longer. As a result, ROS accumulation at the neuroinflammatory sites extends beyond 5 days postoperatively and may persist throughout the duration of the injury.^[^ ^32^, ^38^ ^]^ Consequently, the administration of APTN after 5 days post‐surgery continues to release AD peptide, thereby exerting analgesic effects.

Phagocytosis by macrophages plays a crucial role in regenerative repair following axonal injury. By clearing debris resulting from myelin damage, macrophages create a favorable microenvironment for myelin regeneration.^[^ ^39^ ^]^ However, the accumulation of significant amounts of myelin debris due to severe nerve injury can induce foam‐like transformation of macrophages after phagocytosis, leading to a pro‐inflammatory phenotype that exacerbates neuroinflammation and hinders myelin repair.^[^ ^33^, ^40^ ^]^ Consistently, microglia in the SDH are involved in phagocytosis of myelin from destructed primary afferents, which may result in pain hypersensitivity.^[^ ^33b^ ^]^ Moreover, peripheral nerve injury may cause myelin damage and over‐accumulation of myelin debris. Consequently, phagocytosis of myelin debris can stimulate macrophage polarization toward a pro‐inflammatory M1 phenotype, thus further aggravating nerve injury. RNA‐seq analysis of BMDMs with phagocytosed myelin debris indicated that APTN significantly suppressed the expression of pro‐inflammatory genes (in particular, *Il‐1β*, *Tnf‐α*, *Nos2*, *Nfatc3*, and *Atf2*). Also, APTN treatment reprogrammed myelin debris‐laden BMDMs to the M2 phenotype, by inhibiting the MAPK/NF‐κB signaling pathways and NLRP3 inflammasome activation. These results demonstrated that APTN exerts its anti‐inflammatory effects and promotes nerve regeneration via attenuating local oxidative stress and reprogramming macrophages. Importantly, our results showed that APTN did not significantly reduce macrophage phagocytosis, suggesting that APTN does not impede the phagocytosis and clearance of axonal fragments by macrophages, and therefore does not interfere with the repair process following nerve injury.

Previously, various nanoparticles loaded with NSAIDs, opioids, anesthetics, or neurotoxins have been investigated for the treatment of neuropathic pain,^[^ ^41^ ^]^ showing beneficial outcomes by increasing the bioavailability of different analgesics at target tissues and/or cells. In all these cases, however, nanoparticles were mainly used as biologically inert nanovehicles to achieve site‐specific delivery and/or tailored release of analgesics. Therefore, only anti‐hyperalgesic effects were realized by the loaded analgesics. Importantly, the relationship between the injured nerve and central sensitization, especially the contribution of neuroinflammation to neuropathic pain,^[^ ^7^, ^42^ ^]^ is largely ignored during the design of these nanotherapies. Moreover, nanotherapies capable of effectively treating nerve injury‐induced neuropathic pain are still not fully explored. As for our APTN nanotherapy, in addition to its high targeting efficiency to the injured nerve and tailored release of loaded AD peptide, the nanocarrier itself can resolve neuroinflammation, accelerate nerve regeneration, and shorten pain duration, thereby affording remarkable synergistic anti‐hyperalgesic functions in the treatment of nerve injury‐induced neuropathic pain in rats. Additionally, treatment with APTN is associated with few side effects. Available studies demonstrate that gabapentin co‐inhibits the functions of the α2δ1 and α2δ2 subunits, which are responsible for many side effects of gabapentinoids, including dizziness, somnolence, ataxia, respiratory depression, and even suicidal behaviors.^[^ ^6^, ^37^ ^]^ However, we did not observe any behavioral abnormalities in rats that received single or multiple administrations of AD peptides over a period of up to 28 days. Given that gabapentin analogs pose a risk of inducing depression, our future studies will focus on investigating whether long‐term administration of AD peptides (exceeding one month) can lead to the development of depressive behaviors.

## 4 Conclusion

In summary, we engineered a combination nanotherapy for the precision and effective treatment of nerve injury‐induced neuropathic pain by using an inflammation‐resolving, inflammation‐responsive, and nerve injury‐targeting micellar nanovehicle as well as a calcium channel α2δ1 subunit‐targeting peptide. Besides its targeting delivery and controlled release capacity, the bioactive nanocarrier enabled effective regulation of the neuroinflammatory microenvironment, accelerated neuroregeneration, and reduced central sensitization by suppressing oxidative stress and reprogramming macrophages via inhibiting the MAPK/NF‐κB signaling pathways and NLRP3 inflammasome activation. In combination with a peptide targeting α2δ1‐NMDAR complex, the finally engineered nanoanalgesic realized potent anti‐hyperalgesic activity in rats with nerve injury‐induced neuropathic pain. In addition to neuropathic pain, our nanoanalgesic may serve as a promising therapy for other pain‐related diseases.

## 5 Experimental Section

### Materials

Hexachlorocyclotriphosphazene (HCCP), 4‐(hydroxymethyl) phenylboronic acid pinacol ester (PBE), N‐(tert‐butoxycarbonyl) glycine (Boc‐Gly), 4‐hydroxy‐2,2,6,6‐tetramethylpiperidine‐1‐oxyl (TP), 4‐(dimethylamino) pyridine (DMAP), N, N′‐dicyclohexylcarbodiimide (DCC), sodium hydride (55–65%, NaH), phorbol 12‐myristate 13‐acetate (PMA), 2,2‐diphenyl‐2‐picrylhydrazyl (DPPH), and 4′,6‐diamidino‐2‐phenylindole (DAPI) were purchased from Sigma‐Aldrich (Missouri, U.S.A.). Anhydrous dichloromethane (DCM), anhydrous tetrahydrofuran (THF), triethylamine (TEA), trifluoroacetic acid (TFA), and anhydrous 1, 4‐dioxane (DO) were obtained from the J&K Scientific Ltd (Beijing, China). Cyanine5 NHS ester (Cy5‐NHS) and mPEG‐Amine (Mw = 2000, PEG‐NH_2_) were gained from Ruixi Biological Technology Co., Ltd (Xi'an, China). Penicillin and streptomycin, 2′,7′‐dichlorofluorescin diacetate (DCFH‐DA), cell lysis buffer (P0013), dihydroethidium (DHE), 4′6‐diamidino‐2‐phenylindole (DAPI), BCA protein assay kit (P0012S), protein A+G agarose (Fast Flow, P2055), goat anti‐mouse IgG conjugated with HRP (1:3000, A0216), goat anti‐rabbit IgG conjugated with HRP (1:3000, A0208), goat anti‐mouse IgG with Alexa Fluor 488 (1:400, A0428), and donkey anti‐rabbit IgG with Alexa Fluor 555 (1:400, A0453) antibodies were obtained from Beyotime Biotechnology (Shanghai, China). The cell lines of RAW264.7 (CL‐0190), RSC96 (CL‐0199), and PC‐12 (CL‐0481) were provided by Procell Life Science (Hubei, China). Western HRP substrate (WBKLS0100) and 0.45 µm PVDF transfer membrane (IEVH00005) were purchased from Millipore (Massachusetts, U.S.A.). Total RNA extract reagent (RNAiso Plus, 9108) was provided from TaKaRa (Osaka, Japan). Tris‐HCI SDS‐PAGE at 7.5% or 10% was purchased from Bio‐Rad (California, U.S.A.). Fetal bovine serum (FBS) was acquired from Tianhang Biotechnology Co. Ltd (Zhejiang, China). RPMI 1640 medium was obtained from Gibco (U.S.A.). Amplex Red Hydrogen Peroxide/Peroxidase Assay Kit (A22188) was obtained from Thermo Fisher Scientific (Massachusetts, U.S.A.). Mouse anti IBA1 (1:300, GT10312), rabbit anti‐Arg1 (1:1000, GTX109242), and rabbit anti‐myelin basic protein (MBP, 1:1000, GTX133108) antibody were acquired from GeneTex (Texas, U.S.A.). Mouse anti‐GFAP (1:200, UM500005) antibody was obtained from Origene (U.S.A.). Rabbit anti‐NeuN (1:200, 26975‐1‐AP), rabbit anti‐CD68 (1:200, 25747‐1‐AP), rabbit anti‐MPO (1:300, 22225‐1‐AP), rabbit anti‐iNOS (1:300, 22226‐1‐AP), rabbit anti‐Cacna2d1 (1:100, 27453‐1‐AP), rabbit anti‐GluN1 (1:500, 27676‐1‐AP), rabbit anti‐SCG10 (1:200, 10586‐1‐AP), and rabbit anti‐CD206 (1:500, 18704‐1‐AP) antibodies were provided by Proteintech (Hubei, China). Rabbit anti‐Cacna2d1 (1:800, sc‐271697) antibody was obtained from Santa Cruz Biotechnology (Texas, U.S.A.). Rabbit anti‐ERK1/2 (1:1000, HY‐P80393), anti‐nuclear factor ĸB (NF‐ ĸB) (1:1000, HY‐P80765), anti‐phospho‐NF‐ĸB (1:2000, HY‐P80839), and anti‐NLRP3(1:1000, HY‐P80246) antibodies, granulocyte‐macrophage colony‐stimulating factor (HY‐P7361), cell counting kit‐8 (HY‐K0301), SYBR Green qPCR Master Mix (HY‐K0501A), and RT Master Mix for qPCR II (HY‐K0511A) were acquired from MCE (New Jersey, U.S.A.). Rat anti‐F4/80 (1:500, ab16911) was obtained from Abcam (Cambridge, U.K.). Rabbit anti‐phospho‐ERK1/2 (1:1000, #4370) antibody was provided from Cell Signaling Technology (Massachusetts, U.S.A.). Rabbit anti‐IL1‐β (1:500, A00101‐1), anti‐Pycard (1:500, A00362‐4), anti‐caspase 1 (1:500, BM4291), anti‐β‐actin (1:1000, BM3873) antibody, and all ELISA kits were purchased from BOSTER (Hubei, China).

### Animals

Sprague‐Dawley rats (200 to 240 g) were obtained from the animal experimental center of Army Medical University (Chongqing, China). All animal experiments were approved and endorsed by the Laboratory Animal Welfare and Ethics Committee of the Army Medical University. Animals were accommodated and maintained under specific pathogen‐free (SPF) conditions. All animal experiments were approved by the Animal Ethics and Experimental Committee of the Third Military Medical University (Chongqing, China; No. AMUWEC20210140).

### Surgical Procedures for Peripheral Nerve Injury

To induce neuropathic pain, peripheral nerve injury was accomplished by an operation of transient compression.^[^ ^29^ ^]^ Following anesthesia by intraperitoneal administration of 1% pentobarbital sodium at 4 mL kg^−1^, the animals were positioned laterally. A minor incision was then made at the right hip to expose the sciatic nerve. Subsequently, the nerve was clamped using four vascular clamps, applying compression for 10 min. Once the clamps were removed, the incision was sutured. Postoperatively, the animals were allowed a 3‐day recovery period prior to conducting behavioral assessments.

### Behavioral Assessments of Nociception

Tests of mechanical nociceptive threshold in rats were performed with calibrated von Frey monofilaments on the hind paw plantar surface (Electronic von Frey Anesthesiometer, IITC Life Sciences, U.S.A.). Briefly, rats were adapted to the testing equipment for 30 min before baseline measurement. The experimenter evoked hind paw withdrawal by applying the handheld force transducer with a 0.5 mm^2^ probe to the hind paw of rats, gradually increasing pressure. The pressure intensity was measured and displayed, with the paw withdrawal threshold determined from the average of three measurements. Thermal sensitivity was assessed using a Plantar Test Apparatus (IITC Life Sciences, U.S.A.). The rat was positioned on the glass surface (maintained at 30 °C), and its hindpaws were exposed to radiant heat from a moving light beneath the glass plate. The paw withdrawal latency was recorded when the rat briskly lifted its hindpaws. All behavioral experiments were conducted in a blinded manner.

### Histological Assessments and Immunohistochemical Analyses

To obtain tissue sections, rats were perfused with ice‐cold phosphate‐buffered saline following anesthesia by intraperitoneal injection of 1% pentobarbital sodium at 4 mL kg^−1^. Subsequently, the spinal cords, DRGs, and sciatic nerves were harvested. Following being embedded and placed in an optimal cutting temperature (OCT) compound, 15 µm frozen sections were obtained from fresh frozen sciatic nerves, L5 and L6 spinal cords, and DRG using a cryostat (Leica CM1950, UK). The sections of specified samples were incubated with primary and secondary antibodies. DAPI was utilized for nuclear counterstaining. Immunofluorescence was visualized using a Zeiss LSM880 confocal microscope (Zeiss, UK). The positive cells were quantified by counting in 5–10 random fields (magnification 400×) with ImageJ analysis software (V1.8, NIH).

### TEM Observation of Histological Sections

To prepare ultrathin sections for TEM, the sciatic nerves of rats from experiments were fixed in 2.5% glutaraldehyde for 4 h. Subsequently, the nerves were postfixed with 1% osmium and embedded in resin to facilitate sectioning. TEM images were visualized using a Zeiss LIBRA 200 electron microscope.

### Quantitative Real‐Time Polymerase Chain Reaction (qPCR) Analysis

The steps for the extraction of total RNA and reverse transcription followed the protocol provided by Takara. In brief, total RNA was isolated from sciatic nerves, DRG, and spinal cords, and then reverse transcribed into the complementary DNA (cDNA). qPCR was performed using the SYBR dye in the PCR Detection System (CFX96, Bio‐rad, U.S.A.).

### Enzyme‐Linked Immunosorbent Assay (ELISA)

The sciatic nerves were dissected on ice in pre‐cooled RIPA lysis buffer for 30 min. Subsequently, the dissected tissue was centrifuged at 12000 rpm for 10 min at 4 °C. The protein concentration of the supernatant was tested using the bicinchoninic acid (BCA) method. ELISA analysis was performed to measure the levels of cytokines, including IL‐1β, IL‐6, and TNF‐α.

### Western Blotting Analysis

The protein levels of α2δ1 and NMDARs subunit (GluN1) were quantified by immunoblotting. In brief, spinal cord tissues from rats were collected in pre‐cooled cell lysis buffer, followed by centrifugation at 10 000 g for 20 min at 4 °C. Subsequently, 30 µg total proteins were analyzed using 7.5% Tris‐HCl SDS‐PAGE electrophoresis and transferred onto a PVDF membrane. Primary antibodies and anti‐HRP‐conjugated secondary antibodies were then applied. The membranes were visualized by Western HRP substrate and quantified using the ImageJ analysis (V1.8, NIH). Relative protein levels were normalized to the β‐Actin level.

### Co‐Immunoprecipitation and Western Blotting Analysis

For immunoprecipitation, spinal cord tissues from rats were collected in a pre‐cooled lysis buffer and underwent three freeze‐thaw cycles. Then, the supernatant was incubated overnight with the specified antibodies and gently mixed with protein A/G agarose beads for 6 h. The beads were washed five times by centrifugation with additional cell lysis buffer. Proteins were eluted by adding lysis buffer and boiled in SDS‐PAGE. Subseuqently, samples were processed according to the steps of western blotting analysis.

### RNA‐Sequencing (RNA‐seq) Analysis

The Total RNA from sciatic nerves and DRG of pain rats were isolated using a TRIzol reagent (Invitrogen, U.S.A.). The concentration of total RNA was quantified and assessed using a Bioanalyzer 2100 (Agilent, U.S.A.) with RIN > 7.0. The samples were analyzed by a gene‐sequencing company (LC‐Bio Technology Co., Ltd., Hangzhou, China). Significantly DEGs were identified using the R package DESeq2 with thresholds of a |Log2(fold change, FC)| > 2 and P value < 0.05.

### Synthesis and Characterization of an Inflammation‐Resolving Conjugate

An inflammation‐resolving amphiphile PPT was synthesized by sequential nucleophilic substitution of TP, PEG‐NH_2_, and glycine conjugated PBE (Gly‐PBE) onto HCCP. Specifically, N‐(tert‐butoxycarbonyl) glycine conjugated PBE (Boc‐Gly‐PBE) was first synthesized. Briefly, 25.5 mmol (4.5 g) of Boc‐Gly, 2.55 mmol (320 mg) of DMAP, and 25 mmol (6 g) of PBE were co‐dissolved in 200 mL of DCM. The solution was stirred for 1 h at 0 °C. Subsequently, 25.5 mmol of DCC (5.3 g) was dissolved in 50 mL of DCM and added to the solution. The resulting mixture was filtered after 3 h of stirring and washed with 10% NH_4_Cl, 5% NaHCO_3_, and brine, and then the solvents were removed under vacuum to obtain Boc‐Gly‐PBE. Next, 23 mmol (9 g) Boc‐Gly‐PBE was de‐protected in CF_3_COOH/CH_2_Cl_2_ (1:3) for 3 h. After removing solvents on the vacuum and washing with cold Et_2_O, Gly‐PBE was produced. TP‐conjugated HCCP (HCCP‐TP) was then synthesized. TP (350 mg, 2 mmol) was dissolved in anhydrous THF (10 mL), in which 4 mmol NaH (160 mg) was added in batches under nitrogen protection. At 3 h after the reaction, the mixture was added into 10 mL of anhydrous THF solution containing 2 mmol HCCP (710 mg) at −20 °C, followed by stirring for 3 h again. TLC (hexane: ethyl acetate = 3:1) was used for monitoring the reaction. HCCP‐TP was obtained by purification via chromatography on silica gel.

Then PEG‐conjugated HCCP‐TP (PEG‐HCCP‐TP) was synthesized. Specifically, TEA (168 µL, 1.2 mmol) and HCCP‐TP (320 mg, 0.6 mmol) were added in 20 mL of dry DO at −20 °C, into which 0.5 mmol (1 g) PEG‐NH_2_ was added. After an overnight reaction at room temperature, PEG‐HCCP‐TP was produced. Finally, the PPT amphiphile was synthesized by nucleophilic substitution of PEG‐HCCP‐TP and Gly‐PBE. In this case, Gly‐PBE (1.05 g, 3.6 mmol) and TEA (1 mL) were dissolved in 10 mL of dry DO and added into the above reaction system with PEG‐HCCP‐TP, followed by stirring for 48 h under nitrogen protection at 75 °C. After filtering and concentrating in a vacuum, the residue was dissolved in DCM, precipitated 3 times from cold Et_2_O, and dried to yield the final product PPT.

To synthesize a Cy5‐labeled conjugate (PPE‐Cy5), nucleophilic substitution reaction and amidation reaction of HCCP, Cy5, PEG‐NH_2_, and Gly‐PBE were carried out. N‐Boc‐ethylenediamine (BE)‐conjugated HCCP (HCCP‐BE) was first synthesized. In brief, N‐Boc‐EDA (327 mg, 2 mmol) dissolved in 10 mL of THF containing 10 mL of dry THF containing HCCP (710 mg, 2 mmol) and TEA (615 µL). After stirring for 3 h at −20 °C, HCCP‐BE was filtered, concentrated, and purified through chromatography. PEG‐HCCP‐BE conjugated with four Gly‐PBE units (PP‐BE) was synthesized by replacing HCCP‐TP with HCCP‐BE following the synthetic procedures of PPT. Then PP‐BE was deprotected in CF_3_COOH /CH_2_Cl_2_ (1:3) solution and purified by precipitation from Et_2_O, giving rise to the N‐Boc deprotection product of PP‐BE (PPE). Finally, Cy5‐NHS (0.2 mmol), TEA (0.2 mmol), and PPE (0.2 mmol) were dissolved in DCM and stirred at room temperature for 24 h with protection from light. The mixture was purified by precipitation from Et_2_O, dried under vacuum, and yielded the final product PPE‐Cy5.

For materials characterization, ^1^H NMR spectra were recorded on an Agilent DD2 spectrometer operating at 600 MHz. Fourier transform infrared (FT‐IR) spectra were acquired using a PerkineElmer FT‐IR spectrometer (100S). Matrix‐assisted laser desorption/ionization time‐of‐flight (MALDI‐TOF) mass spectrometry was conducted on a Bruker Daltonics ultrafleXtreme MALDI‐TOF/TOF mass spectrometer in linear mode.

### Fabrication and Characterization of PPT Nanomicelles with or without a Peptide Drug

PPT nanomicelles (PPTN) were prepared by dissolving PPT directly in deionized water. To fabricate Cy5‐labeled PPT nanomicelles (i.e., PPTN‐Cy5), PPT and PPE‐Cy5 at a weight ratio of 9:1 were dissolved directly into aqueous solutions. PPT nanomicelles loaded with the AD peptide (i.e., APTN) were fabricated through nanoprecipitation and self‐assembly. Specifically, 100 mg of PPT and 4 mg of AD peptide were dissolved in 20 mL of deionized water and 0.5 mL of methanol, respectively. The organic solution containing the AD peptide was then added dropwise to 20 mL of water containing PPT while gently stirring. After dialysis in deionized water at room temperature for 12 h, APTN was harvested by lyophilization.

The content of AD peptide in APTN was determined by high‐performance liquid chromatography (Shimadzu, Japan). The size distribution and ζ‐potential values of both PPTN and APTN were determined using dynamic light scattering (Malvern Zetasizer NanoZS, Netherlands). The transmission electron microscopy (TEM) images of PPTN and APTN were visualized using a LIBRA 200 electron microscope (Zeiss, Germany).

### ROS‐Scavenging Activities of PPT

The free radical scavenging capacity of PPT amphiphile was first examined. Briefly, 100 µL of methanol with varying concentrations of PPT was mixed with 100 µL methanol solution of DPPH• (100 µg mL^−1^), and then incubated at room temperature for different durations. The absorbance at 520 nm was tested at different time points by a microplate reader (Molecular Devices, U.S.A.), and the DPPH• scavenging values were calculated. To assess the hydrogen peroxide scavenging capability of PPT, 1 mL of 1 mM H_2_O_2_ was mixed with 100 µL of methanol containing different concentrations of PPT and incubated for 2 h at 37 °C. The remaining H_2_O_2_ was measured by the Hydrogen Peroxide Assay Kit, and the H_2_O_2_ scavenging capability was identified by examining the absorbance at 560 nm.

### Hydrolysis of PPT in the Presence of H_2_O_2_

To examine hydrolysis of PPT in H_2_O_2_, PPT was incubated with an aqueous solution containing 10 mM H_2_O_2_ at 37 °C for 24 h. After complete hydrolysis, the contents of TP and p‐(hydroxymethyl)phenol (HMP) in the hydrolyzed samples were determined by an ultra‐performance liquid chromatography (UPLC) system (Waters, U.S.A.) using a methanol‐water eluent (20:80, v:v). The UPLC experiments were measured at 254 nm at a flow rate of 0.5–1 mL min^−1^.

### Cell Culture

PC12 neuron‐like cells, RSC96 Schwann cells, and RAW264.7 macrophages were cultured using RPMI‐1640 complete medium. BMDMs were mechanically harvested from male C57B/L6 mice and cultured in RPMI‐1640 complete medium.

### In Vitro Antioxidant and Anti‐Inflammatory Activities of PPTN

RAW264.7 cells (3 × 10^5^ cells per well) were treated with PMA (100 ng mL^−1^) and incubated overnight. Then, different doses of PPTN (10–100 µg mL^−1^) were added for 4 h. The cells were harvested, washed by PBS, and incubated with DCFH‐DA (a ROS‐sensitive fluorescent dye) at 37 °C for 30–60 min. Finally, the fluorescence intensity of the cells was determined using flow cytometry and a confocal laser scanning microscope (CLSM).

### In Vivo Targeting Capability of PPTN and APTN

PPTN‐Cy5 was administered intravenously at a dose of 10 mg kg^−1^ to rats with neuropathic pain. Rats were euthanized at specified time points post‐administration, and their brains, spinal cords, and sciatic nerves were collected under ice‐cold conditions. Ex vivo fluorescence imaging was conducted and analyzed using an In Vivo Imaging System (Newton, Viber Bio Imaging, France).

### Analgesic Effects of AD Peptide, PPTN, and APTN in Rats with Neuropathic Pain

Neuropathic pain was induced in rats through peripheral nerve injury. Post‐injury, the rats were randomly assigned into four groups (*n* = 5) and were administered intravenously with saline, AD peptide (0.4 mg kg^−1^), PPTN (10 mg kg^−1^), or APTN (10 mg kg^−1^). To evaluate acute anti‐hyperalgesic efficacy, a single injection was administered. Mechanical nociceptive thresholds were measured every 30 min immediately after administration and continued for 7 h post‐injection. For assessing the long‐term analgesic effects, three injections were administered on days 1, 3, and 5 post‐injury. Behavioral tests were performed daily, starting on day 1 post‐injury and continuing until day 30 post‐surgery. For behavioral tests, the previously described procedures were followed.

In addition, co‐immunoprecipitation and western blotting analysis were conducted to detect the binding interaction between α2δ1and NMDAR. On day 14 post‐injury, rats from each group were euthanized, and spinal cord tissues were harvested. The co‐immunoprecipitation and western blotting procedures were then performed following the previously described protocols. For immunofluorescence analysis of tissue sections, peripheral nerves from rats in each group were collected on day 14 post‐injury. Subsequent histological assessments and immunohistochemical analyses were carried out in accordance with the methods detailed earlier.

### In Vivo Toxicity Tests

Preliminary in vivo safety studies were developed in healthy rats. Rats were randomly divided into three groups. The control group received i.v. administration of saline, while the other two groups were treated with APTN at doses of 50 or 100 mg kg^−1^ via i.v. injection. The body weight and behaviors of rats were monitored at predetermined time intervals post‐injection. After 14 days, the rats were euthanized, and blood samples were collected. The major organs were harvested, weighed, and fixed for histological analysis.

### Statistical Analysis

All quantitative data are presented as mean ± standard deviation (SD). One‐way analysis of variance (ANOVA) and two‐tailed Student's t‐test, followed by Tukey's honestly significant difference tests, were performed for comparisons between the two groups. Two‐way ANOVA followed by Tukey's honestly significant difference test, was used to compare the behavioral data at different time points among more than two groups. A statistically significant difference was defined as a P value less than 0.05.

## Conflict of Interest

The authors declare no conflict of interest.

## Author Contributions

W.K.W. and Y.W. contributed equally to this work. J.X.Z. and Y.Z. conceived the project. J.X.Z., W.K.W., and Y.W. designed the experiments. W.K.W., Y.W., X.L.H., P.W., L.L.L., Y.Z., and Y.H.C. performed the experiments. W.K.W., Y.W., Z.Y.C., and C.Q.L. analyzed and interpreted the data. J.X.Z., W.K.W., Y.W., and Y.Z. wrote the manuscript. Supervision was provided by J.X.Z. and Y.Z. All authors discussed the results and reviewed the manuscript.

## Supporting information



Supporting Information

## Data Availability

The data that support the findings of this study are available from the corresponding author upon reasonable request.

## References

[1] a) S. P. Cohen , L. Vase , W. M. Hooten , Lancet 2021, 397, 2082; 34062143 10.1016/S0140-6736(21)00393-7

[2] S. R. A. Alles , P. A. Smith , Pharmacol. Rev. 2018, 70, 315. 29500312 10.1124/pr.117.014399

[3] K. Bannister , J. Sachau , R. Baron , A. H. Dickenson , Ann. Rev. Pharmacol. Toxicol. 2020, 60, 257. 31914896 10.1146/annurev-pharmtox-010818-021524

[4] N. B. Finnerup , R. Kuner , T. S. Jensen , Physiol. Rev. 2021, 101, 259. 32584191 10.1152/physrev.00045.2019

[5] S. Mathieson , C. C. Lin , M. Underwood , S. Eldabe , BMJ 2020, 369, m1315. 32345589 10.1136/bmj.m1315

[6] a) C. D. Williams , Z. Al‐Jammali , M. C. Herink , Drugs 2023, 83, 37; 36529848 10.1007/s40265-022-01810-3

[7] a) C. Sommer , M. Leinders , N. Uceyler , Pain 2018, 159, 595; 29447138 10.1097/j.pain.0000000000001122

[8] a) X. Gao , H. K. Kim , J. M.o Chung , K. Chung , Pain 2007, 131, 262; 17317010 10.1016/j.pain.2007.01.011 PMC2048490

[9] X. Yu , H. Liu , K. A. Hamel , M. G. Morvan , S. Yu , J. Leff , Z. Guan , J. M. Braz , A. I. Basbaum , Nat. Commun. 2020, 11, 264. 31937758 10.1038/s41467-019-13839-2 PMC6959328

[10] a) J. Wei , W. Su , Y. Zhao , Z. Wei , Y. Hua , P. Xue , X. Zhu , Y. Chen , G. Chen , J. Neuroinflamm. 2022, 19, 32; 10.1186/s12974-022-02405-1 PMC8809034 35109876

[11] F. Bonelli , I. Demirsoy , R. M. Lasagni Vitar , P. Fonteyne , G. Ferrari , Ocul. Surf. 2023, 30, 92. 37690516 10.1016/j.jtos.2023.09.004

[12] a) T. Hanke , D. Merk , D. Steinhilber , G. Geisslinger , M. Schubert‐Zsilavecz , Pharmacol. Ther. 2016, 157, 163; 26627986 10.1016/j.pharmthera.2015.11.011

[13] a) S. Ramadan , T. Li , W. Yang , J. Zhang , Z. Rashidijahanabad , Z. Tan , N. Parameswaran , X. Huang , ACS Cent. Sci. 2020, 6, 913; 32607438 10.1021/acscentsci.9b01199 PMC7318065

[14] a) K. Hu , L. Zhong , W. Lin , G. Zhao , W. Pu , Z. Feng , M. Zhou , J. Ding , J. Zhang , ACS Nano 2024, 18, 6650; 38369729 10.1021/acsnano.4c00120

[15] S. P. Cohen , J. Mao , BMJ 2014, 348, f7656. 24500412 10.1136/bmj.f7656

[16] B. Botz , K. Bolcskei , Z. Helyes , Wiley Interdiscip. Rev. Nanomed. Nanobiotechnol. 2017, 9, e1427. 10.1002/wnan.1427 27576790

[17] M. F. Seidel , T. Hugle , B. Morlion , M. Koltzenburg , V. Chapman , A. MaassenVanDenBrink , N. E. Lane , S. Perrot , W. Zieglgansberger , Exp. Neurol. 2022, 356, 114108. 35551902 10.1016/j.expneurol.2022.114108

[18] C. B. Burness , P. L. McCormack , Drugs 2016, 76, 123. 26666418 10.1007/s40265-015-0520-9

[19] M. D. Boada , T. J. Martin , R. Parker , T. T. Houle , J. C. Eisenach , D. G. Ririe , Pain 2020, 161, 949. 32040074 10.1097/j.pain.0000000000001781 PMC7166146

[20] H. D. Gilchrist , B. L. Allard , D. A. Simone , Pain 1996, 67, 179. 8895246 10.1016/0304-3959(96)03104-1

[21] a) E. Jung , J. Lee , L. Jeong , S. Park , M. Lee , C. Song , D. Lee , Biomaterials 2019, 192, 282; 30458363 10.1016/j.biomaterials.2018.11.022

[22] C. S. Wilcox , Pharmacol. Ther. 2010, 126, 119. 20153367 10.1016/j.pharmthera.2010.01.003 PMC2854323

[23] L. Shi , J. Zhang , M. Zhao , S. Tang , X. Cheng , W. Zhang , W. Li , X. Liu , H. Peng , Q. Wang , Nanoscale 2021, 13, 10748. 34132312 10.1039/d1nr02065j

[24] H. Maeda , J. Drug Target. 2017, 25, 781. 28988499 10.1080/1061186X.2017.1365878

[25] a) S. Ruan , Y. Zhou , X. Jiang , H. Gao , Adv. Sci. 2021, 8, 2004025; 10.1002/advs.202004025 PMC8097396 33977060

[26] D. Furtado , M. Bjornmalm , S. Ayton , A. I. Bush , K. Kempe , F. Caruso , Adv. Mater. 2018, 30, 1801362. 10.1002/adma.201801362 30066406

[27] a) J. M. Boggs , Cell. Mol. Life Sci. 2006, 63, 1945; 16794783 10.1007/s00018-006-6094-7 PMC11136439

[28] a) E. Bourinet , C. Altier , M. E. Hildebrand , T. Trang , M. W. Salter , G. W. Zamponi , Physiol. Rev. 2014, 94, 81; 24382884 10.1152/physrev.00023.2013

[29] W. Wang , X. Huang , Y. Zhang , J. Wu , Y. Wang , L. Li , J. Zhang , Y. Zhou , Pain Physician 2024, 27, E131. 38285045

[30] a) J. Chen , L. Li , S. R. Chen , H. Chen , J. D. Xie , R. E. Sirrieh , D. M. MacLean , Y. Zhang , M. H. Zhou , V. Jayaraman , H. L. Pan , Cell Rep. 2018, 22, 2307; 29490268 10.1016/j.celrep.2018.02.021 PMC5873963

[31] L. Li , S. R. Chen , M. H. Zhou , L. Wang , D. P. Li , H. Chen , G. Lee , V. Jayaraman , H. L. Pan , Cell Rep. 2021, 36, 109396. 34289359 10.1016/j.celrep.2021.109396 PMC8353586

[32] K. Inoue , M. Tsuda , Nat. Rev. Neurosci. 2018, 19, 138. 29416128 10.1038/nrn.2018.2

[33] a) L. M. Milich , C. B. Ryan , J. K. Lee , Acta Neuropathol. 2019, 137, 785; 30929040 10.1007/s00401-019-01992-3 PMC6510275

[34] a) J. A. Martina , E. Jeong , R. Puertollano , EMBO Rep. 2023, 24, e55472; 36507874 10.15252/embr.202255472 PMC9900348

[35] J. Wu , Y. Han , H. Xu , H. Sun , R. Wang , H. Ren , G. Wang , Sci. Adv. 2023, 9, eadi8343. 37801503 10.1126/sciadv.adi8343 PMC10558133

[36] a) N. P. Goncalves , C. B. Vaegter , H. Andersen , L. Ostergaard , N. A. Calcutt , T. S. Jensen , Nat. Rev. Neurol. 2017, 13, 135; 28134254 10.1038/nrneurol.2016.201 PMC7391875

[37] K. E. Evoy , S. Sadrameli , J. Contreras , J. R. Covvey , A. M. Peckham , M. D. Morrison , Drugs 2021, 81, 125. 33215352 10.1007/s40265-020-01432-7

[38] G. Chen , Y. Q. Zhang , Y. J. Qadri , C. N. Serhan , R. R. Ji , Neuron 2018, 100, 1292. 30571942 10.1016/j.neuron.2018.11.009 PMC6312407

[39] P. Chen , X. Piao , P. Bonaldo , Acta Neuropathol. 2015, 130, 605. 26419777 10.1007/s00401-015-1482-4

[40] a) X. Wang , K. Cao , X. Sun , Y. Chen , Z. Duan , L. Sun , L. Guo , P. Bai , D. Sun , J. Fan , X. He , W. Young , Y. Ren , Glia 2015, 63, 635; 25452166 10.1002/glia.22774 PMC4331228

[41] a) D. Bhansali , S. L. Teng , C. S. Lee , B. L. Schmidt , N. W. Bunnett , K. W. Leong , Nano Today 2021, 39, 101223; 34899962 10.1016/j.nantod.2021.101223 PMC8654201

[42] a) S. Rotshenker , J. Neuroinflamm. 2011, 8, 109; 10.1186/1742-2094-8-109 PMC3179447 21878125

