# Supplementary material for: Pathophysiology‐Directed Engineering of a Combination Nanoanalgesic for Neuropathic Pain
Source: Adv Sci (Weinh). 2024 Dec 24;12(8):2405483. doi: 10.1002/advs.202405483 (PMC11848598; doi:10.1002/advs.202405483)
Supplement: Supplementary file 1 — Supporting Information [file ADVS-12-2405483-s001.docx]

Supporting Information

**Pathophysiology-Directed Engineering of a Combination Nanoanalgesic for Neuropathic Pain**

*Wenkai Wang, Yan Wang, Xinle Huang, Peng Wu, Lanlan Li, Yang Zhang, Yihui Chen, Zhiyu Chen, Changqing Li, Yue Zhou*, Jianxiang Zhang**

W. K. Wang, X. L. Huang, Y. Zhang, C. Q. Li, Y. Zhou

Department of Orthopedics, Xinqiao Hospital, Third Military Medical University (Army Medical University), Chongqing 400037, P. R. China

E-mail: happyzhou@vip.163.com

W. K. Wang

Department of Orthopedics, General Hospital of PLA Xizang Military Area Command, Lhasa 850007, P. R. China

Y. Wang, P. Wu, L. L. Li, J. X. Zhang

Department of Pharmaceutics, College of Pharmacy, Third Military Medical University (Army Medical University), Chongqing 400038, P. R. China

E-mail: jxzhang@tmmu.edu.cn

Y. Wang

War Trauma Medical Center, State key Laboratory of Trauma, Burns and Combined injury, Army Medical Center, Daping Hospital, Third Military Medical University (Army Medical University), Chongqing 400038, P. R. China

X. L. Huang

Department of Orthopedics, The Second Naval Hospital of Southern Theater Command, Sanya 572000, P. R. China

P. Wu

School of Pharmacy, Hanzhong Vocational and Technical College, Hanzhong 723002, P. R. China

Y. H. Chen

Department of General Surgery, Xinqiao Hospital, Third Military Medical University (Army Medical University), Chongqing 400037, P. R. China

Z. Y. Chen

Department of Orthopedics, The First Affiliated Hospital, Chongqing Medical University, Chongqing 400016, P. R. China

J. X. Zhang

State Key Laboratory of Trauma and Chemical Poisoning, Third Military Medical University (Army Medical University), Chongqing 400038, P. R. China

J. X. Zhang

Yu-Yue Pathology Scientific Research Center, Chongqing 400039, P. R. China

**Table S1.** Physicochemical properties of different AD peptide-containing PPT nanomicelles. The data are expressed as means ± SD (n = 3).

| Weight ratio of PPT/AD peptide | 25:1 | 10:1 | 5:1 |
| --- | --- | --- | --- |
| Diameter (nm) | 71.8 ± 0.9 | 62.9 ± 4.2 | 35.7 ± 3.1 |
| Polydispersity index | 0.2 ± 0.01 | 0.2 ± 0.03 | 0.2 ± 0.02 |
| ζ-Potential (mV) | 11.8 ± 0.3 | 15.2 ± 0.5 | 20.9 ± 1.4 |
| Entrapment efficiency (%) | 83.0 ± 6.9 | 21.5 ± 3.2 | 5.3 ± 1.6 |
| AD peptide loading (μg/mg) | 33.2 ± 1.2 | 21.5 ± 0.9 | 10.7 ± 0.7 |

**
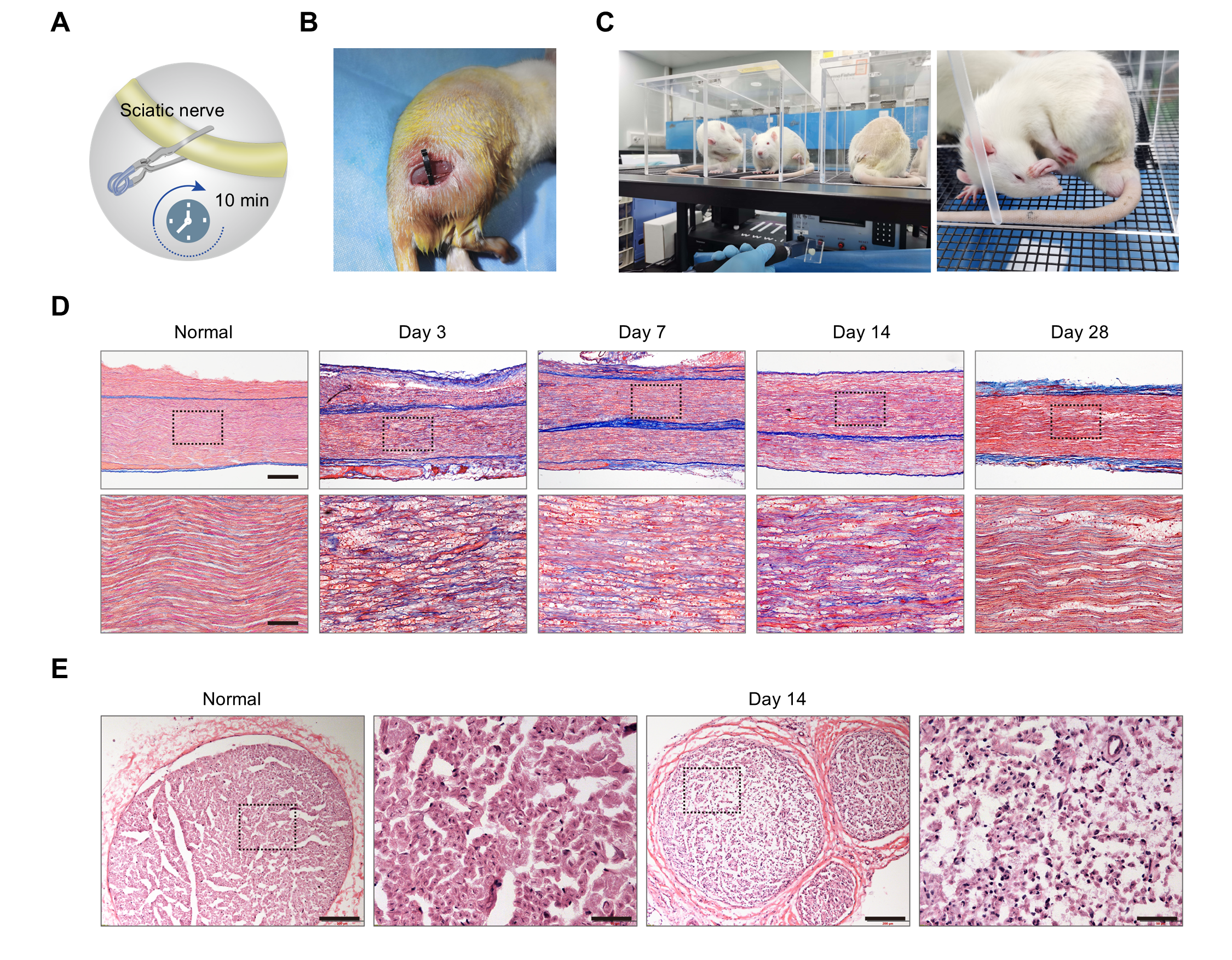
**

**Figure S1.** Establishment and typical characteristics of a rat model with peripheral nerve injury. (A) Schematic illustration of the surgical procedures for inducing nerve injury. The sciatic nerve was clipped by vascular clamps for 10 min. (B) A photograph illustrates the surgical procedure. (C) Photographs showing behavioral tests of rats with von Frey monofilaments. (D) Masson staining of the longitudinal sections of injured nerves at different time points after surgery. Regions in the black squares are magnified and shown in the lower panel. Scale bars: 500 μm (upper), and 200 μm (lower). (E) H&E-stained cross-sections of injured nerves at day 14 after surgery. Regions in the black squares are magnified and illustrated in the right panel. Scale bars: 200 μm (upper), and 50 μm (lower).


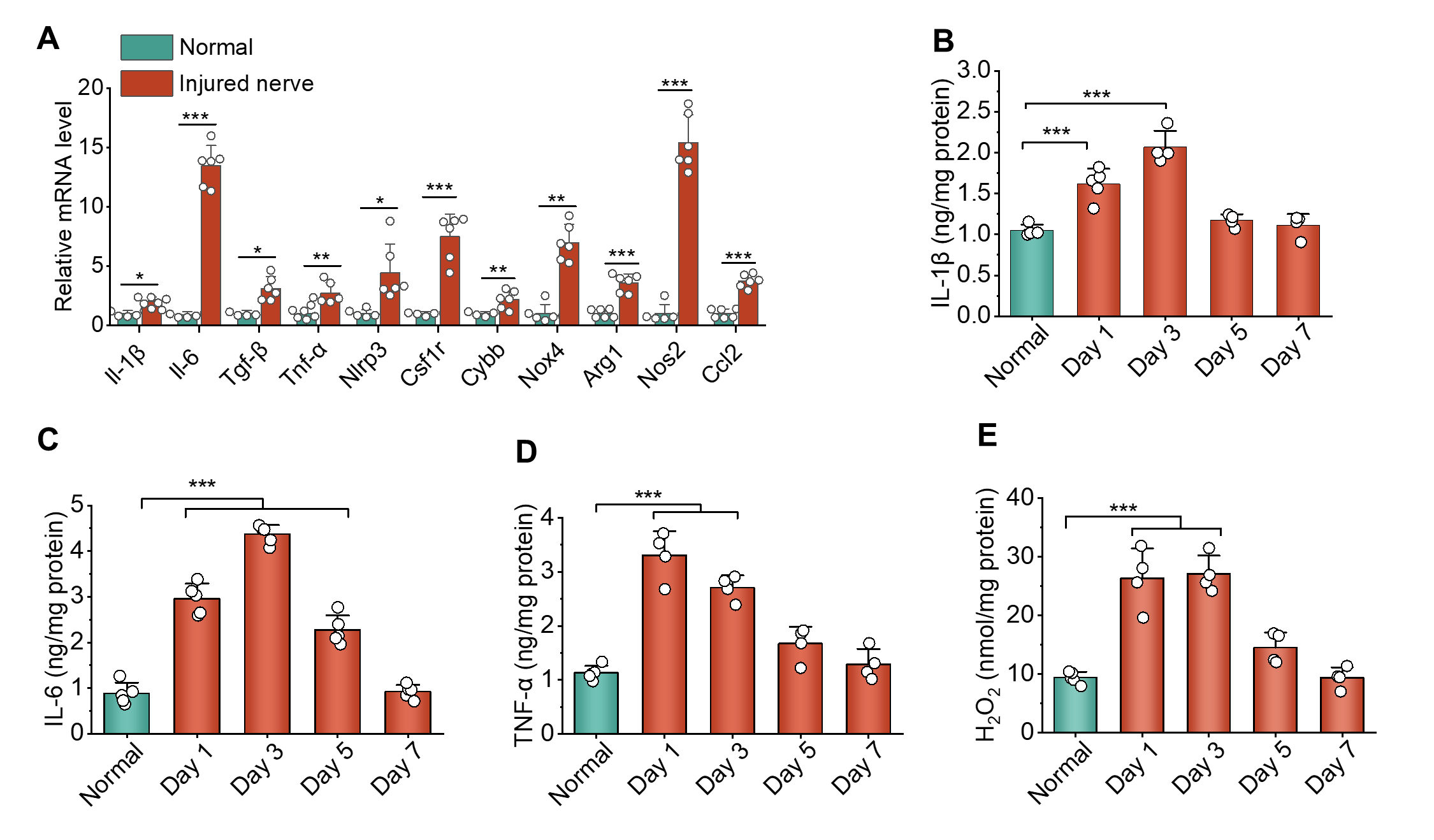


**Figure S2.** The expression of inflammatory cytokines and the levels of ROS in the injured nerve. (A) qPCR analysis of typical inflammatory cytokines in the normal and injured sciatic nerves (n = 6). (B-D) The expression levels of TNF-α, IL-1β, and IL-6 in the injured nerve at different time points after surgery (n = 4-5). (E) The levels of H_2_O_2_ in the injured nerve at different time points after surgery (n = 4). The data are expressed as means ± SD. *p < 0.05, **p < 0.01, ***p < 0.001.


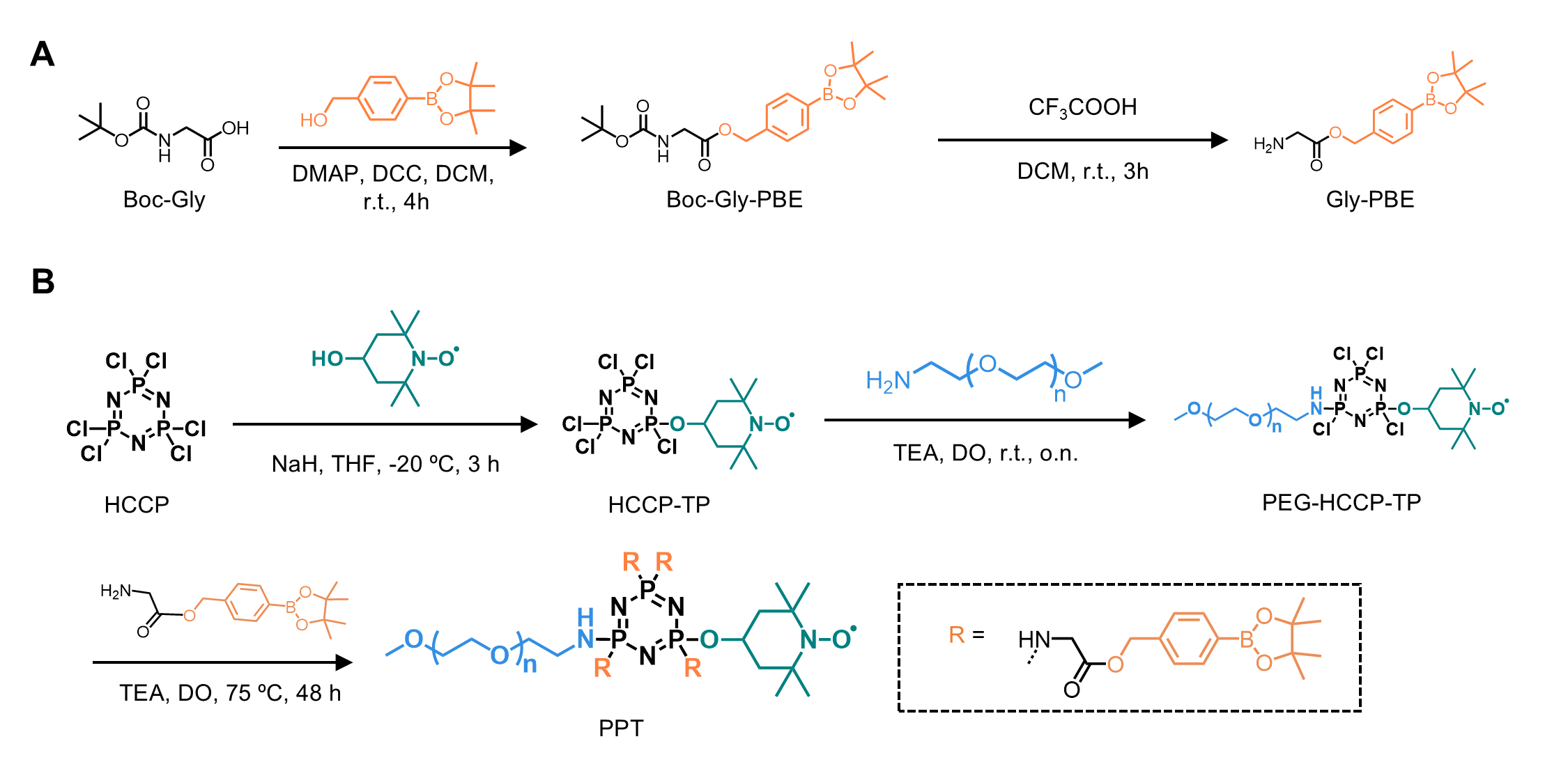


**Figure S3.** Synthetic routes of the PPT amphiphile. (A) The synthetic route of Gly-PBE by the reaction of esterification and N-Boc deprotection. PBE, 4-(hydroxymethyl) phenylboronic acid pinacol ester; Boc-Gly, N-(tert-Butoxycarbonyl) glycine; DMAP, 4-dimethylaminopyridine; DCC, N, N′-dicyclohexylcarbodiimide; DCM, anhydrous dichloromethane. (B) The synthetic route of PPT. TP, PEG-NH_2_, and Gly-PBE units were conjugated onto HCCP by consecutive nucleophilic substitution reactions. HCCP, hexachlorocyclotriphosphazene; NaH, sodium hydride; THF, anhydrous tetrahydrofuran; TP, 4-hydroxy-2,2,6,6-tetramethylpiperidine-1-oxyl; DO, anhydrous 1, 4-dioxane; r.t., room temperature; o.n., overnight.


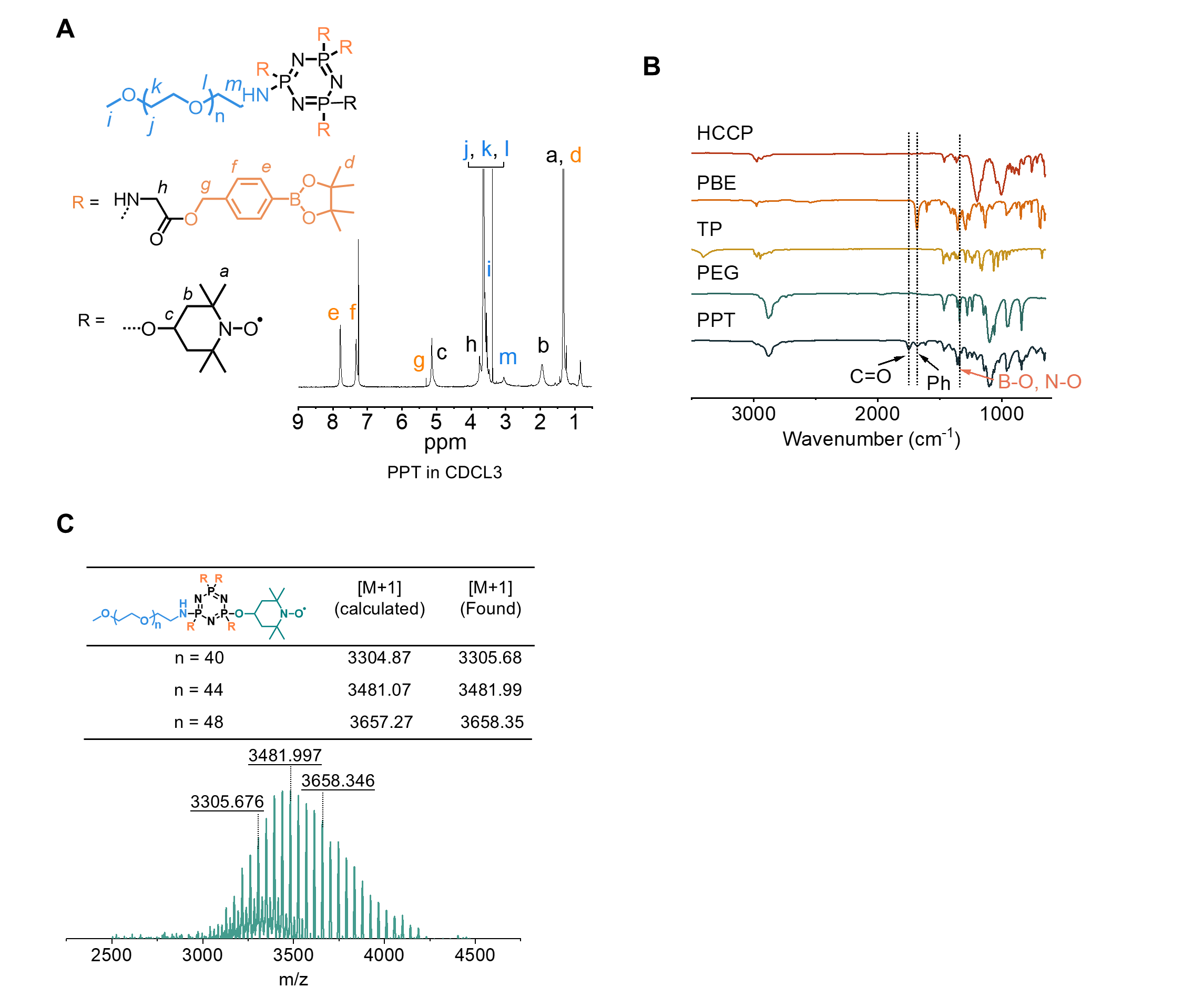


**Figure S4.** Characterization of the PPT amphiphile. (A) The ^1^H NMR spectrum of PPT in CDCl_3_. (B) FT-IR spectra of different materials. (C) The MALDI-TOF mass spectrum of PPT.


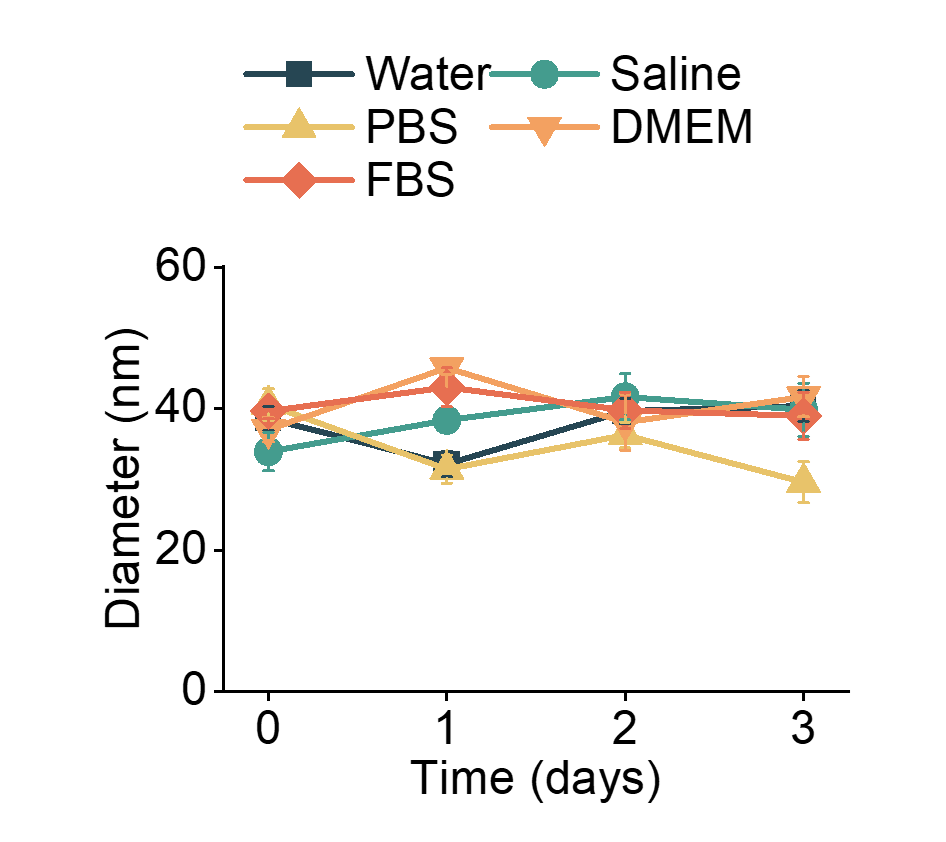


**Figure S5.** Changes of the mean diameter of PPT after incubation in different solutions for 3 days. The data are expressed as means ± SD (n = 5).


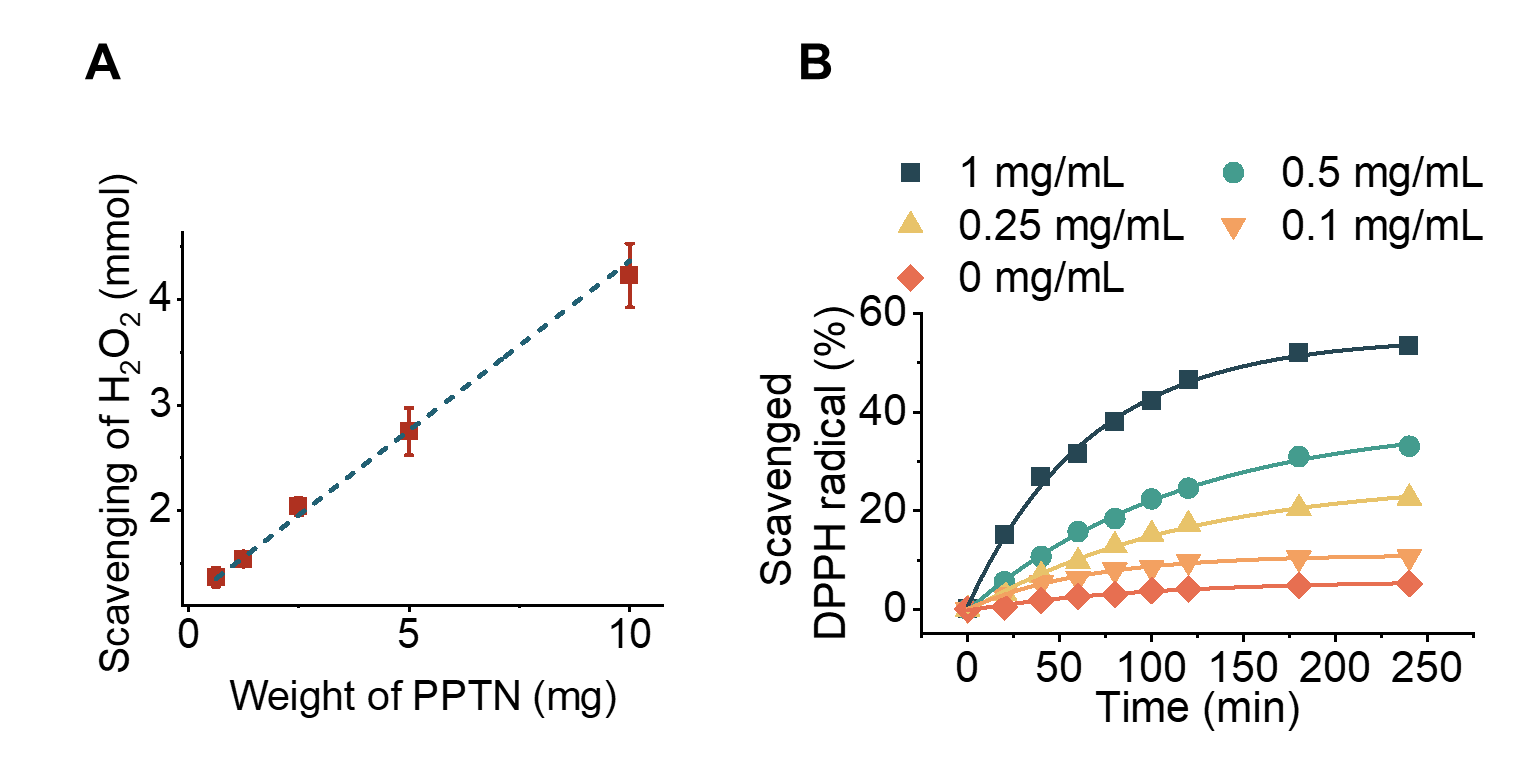


**Figure S6.** Scavenging of ROS by PPT. (A) Dose-dependent elimination of H_2_O_2_ by PPT. (B) Time-dependent elimination of the DPPH radical by different doses of PPT. The data are expressed as means ± SD (n = 3).


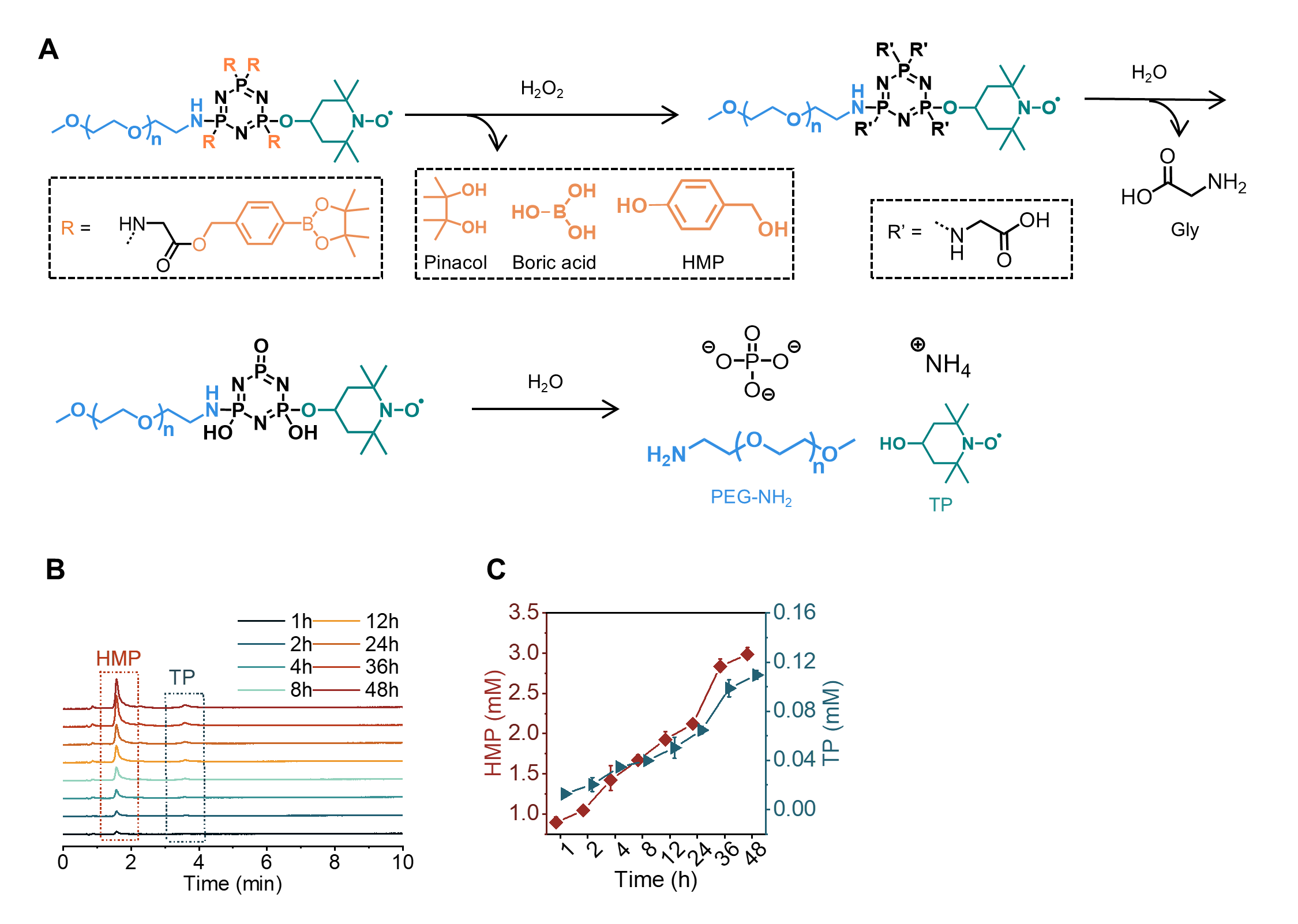


**Figure S7.** Hydrolysis of PPT. (A) The mechanisms underlying H_2_O_2_-triggered hydrolysis of PPT. HMP, p-(hydroxymethyl)phenol; Gly, glycine. (B-C) HPLC curves (B) and quantification of HMP and TP (C) indicating the time-dependent hydrolysis of PPT at 1 mM H_2_O_2_.

**
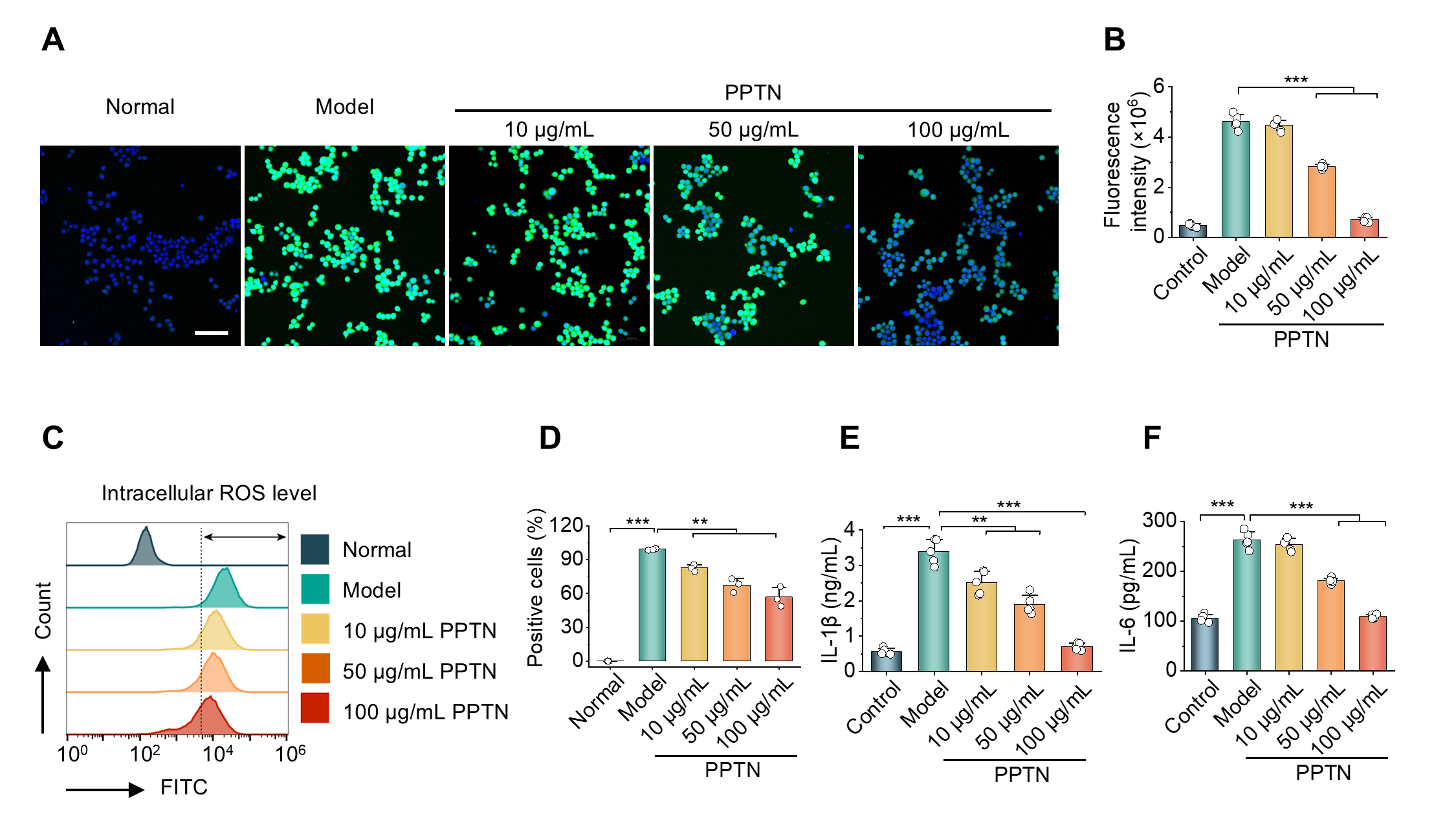
**

**Figure S8.** In vitro antioxidative and anti-inflammatory effects of PPTN in macrophages. (A-B) Fluorescence microscopy images (A) and quantitative data (B) showing the intracellular ROS generation in RAW264.7 cells (n = 5). Scale bar, 200 μm. (C-D) Flow cytometric profiles (C) and quantification (D) of intracellular ROS in RAW264.7 cells (n = 3). RAW264.7 cells stimulated with PMA were used for investigating therapeutic effects of PPTN. Cells were incubated with 200 ng/mL PMA and treated with different doses of PPTN for 6 h. The intracellular ROS level was detected with a fluorescent probe DCFH-DA. (E-F) The expression levels of IL-1β (E) and IL-6 (F) in RAW264.7 cells after treatment with different doses of PPTN (n = 5). The data are expressed as means ± SD. **p < 0.01, ***p < 0.001.


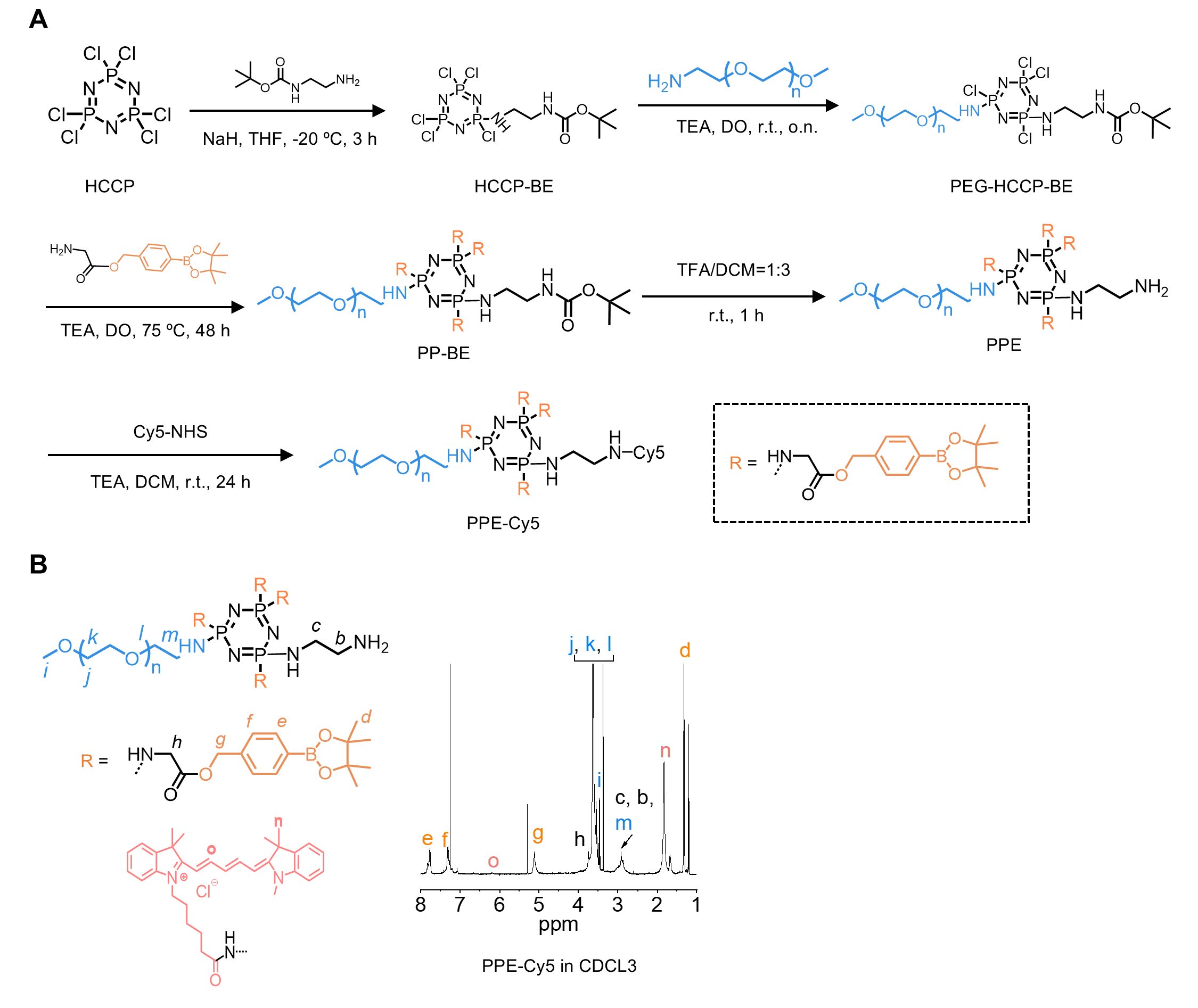


**Figure S9.** Synthesis and characterization of the PPE-Cy5 amphiphile. (A) The synthetic route of PPE-Cy5. HCCP was conjugated with PEG-NH_2_, Gly-PBE, and Cy5 units by nucleophilic substitution and condensation reactions. HCCP-BE, HCCP conjugated with N-Boc-ethylenediamine (BE); PEG-HCCP-BE, HCCP-BE conjugated with PEG-NH_2_; PP-BE, PEG-HCCP-BE conjugated with Gly-PBE; PPE, the N-Boc deprotection product of PP-BE; PPE-Cy5, the final product of Cy5-conjugated PPE. (B) The ^1^H NMR spectrum of Cy5-conjugated PPE (PPE-Cy5).

**
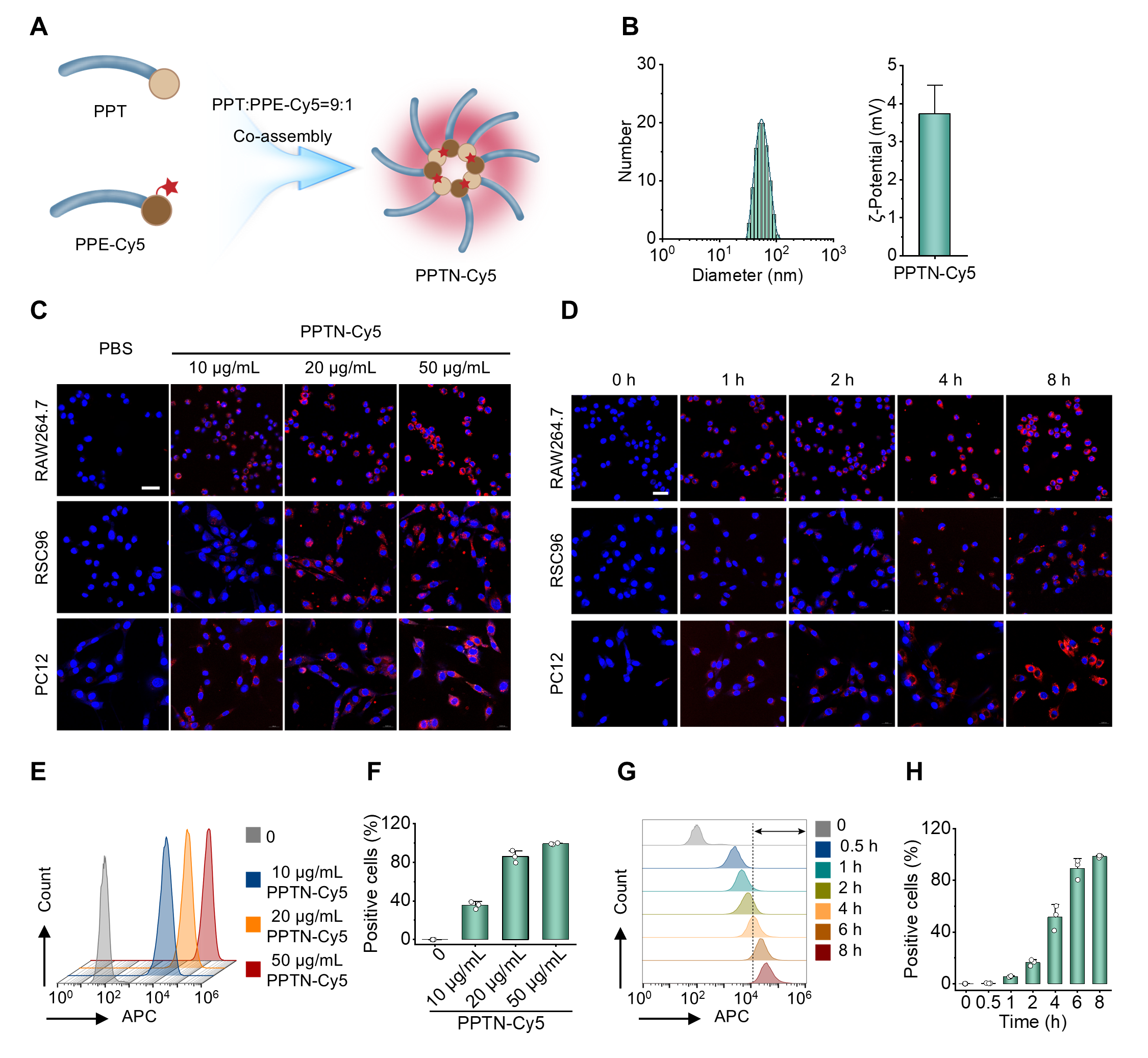
**

**Figure S10.** Cellular uptake of Cy5-labeled PPTN in RAW264.7 macrophages, RSC96 Schwann cells, and PC12 neuron-like cells. (A) A sketch shows engineering of Cy5-labeled PPTN (PPTN-Cy5). (B) The size distribution profile and ζ-potential of PPTN-Cy5. (C) Fluorescence images showing dose-dependent internalization of PPTN-Cy5 in RAW264.7, RSC96, and PC12 cells at 2 h after incubation. (D) Fluorescence images showing time-dependent internalization of PPTN-Cy5 at 10 μg/mL in RAW264.7, RSC96, and PC12 cells. (E-F) Flow cytometric profiles (E) and quantification (F) of dose-dependent cellular uptake of PPTN-Cy5 in RAW264.7 cells after 2 h of incubation. (G-H) Typical flow cytometric curves (G) and quantitative analysis (H) of time-dependent cellular uptake of PPTN-Cy5 at 10 μg/mL in RAW264.7 cells. Scale bars in (C and D), 50 μm. The data are expressed as means ± SD (n = 3).


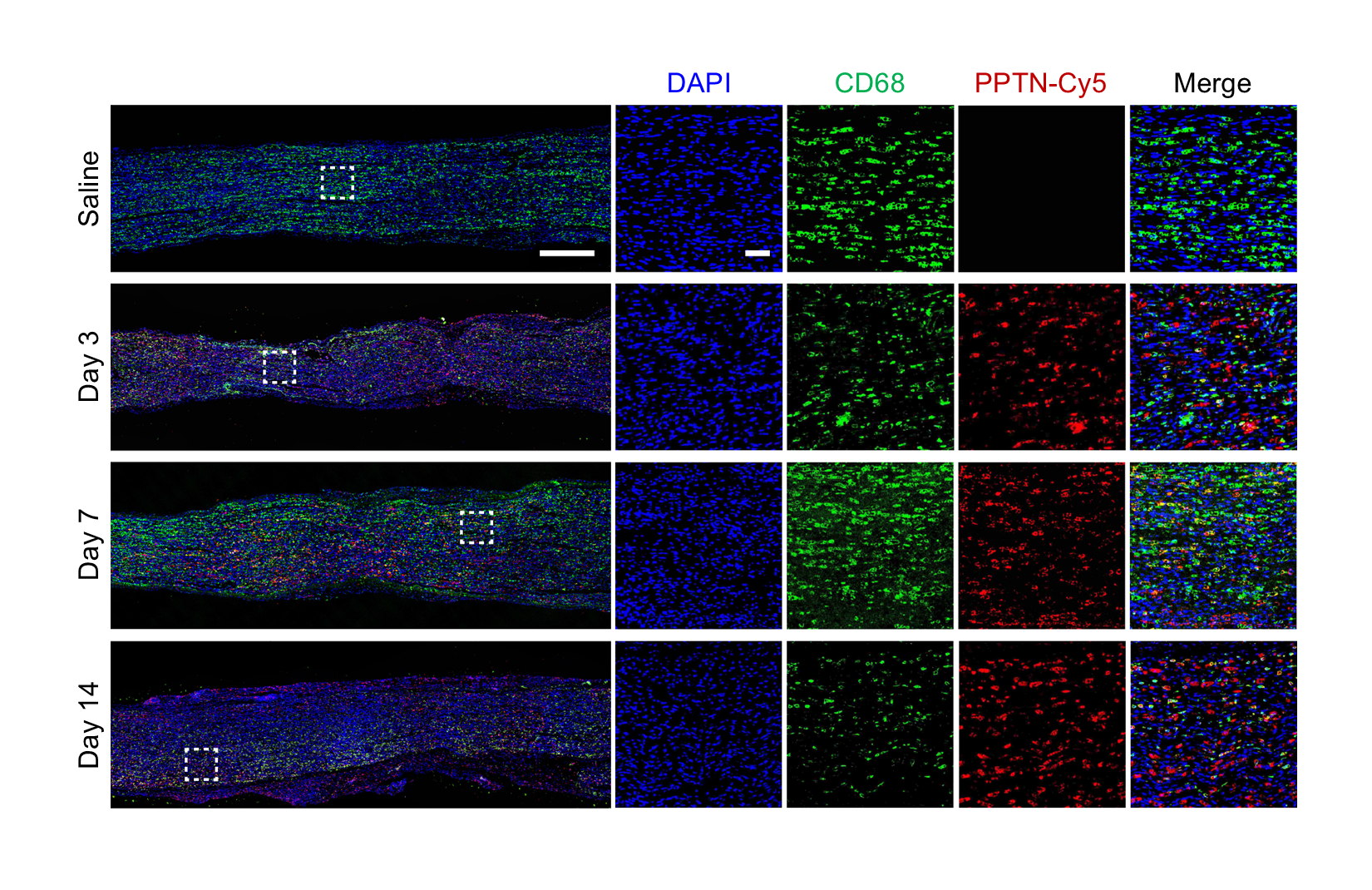


**Figure S11.** Immunofluorescence images indicate co-localization of PPTN-Cy5 with CD68^+^ macrophages in longitudinal sections of sciatic nerves at different time points after injury. These images show additional regions for magnification from the sections of each group corresponding to Figure 3I. The right panel images show magnified regions in the white squares. All tissues were isolated at 6 h after i.v. injection. Scale bars, 200 μm (left) and 20 μm (right).


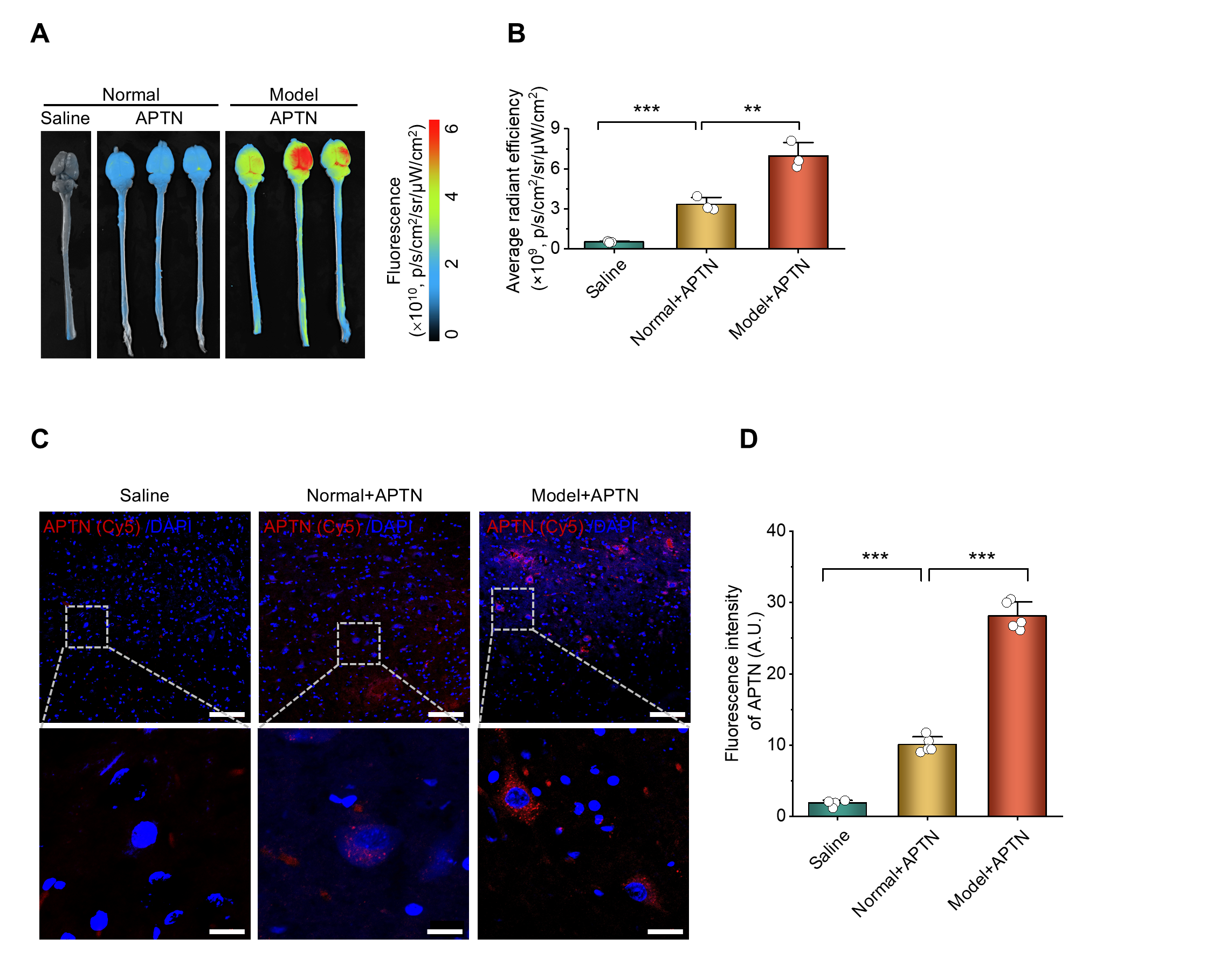


**Figure S12.** The CNS targeting capability of APTN in rats under physiological and pathological conditions. (A-B) Ex vivo fluorescence images (A) and quantitative data (B) show the accumulation of APTN in the CNS of rats at 12 h after i.v. injection. Saline, healthy rats injected with saline; Normal+APTN, healthy rats injected with APTN-Cy5; Model+APTN, rats with peripheral nerve injury injected with APTN-Cy5. (C-D) Fluorescence images (C) and quantitative data (D) show distribution of APTN in spinal cord cross-sections in different groups. Images in the lower panels illustrate the magnified region in the white square. Tissues were isolated at 12 h after i.v. injection. Scale bars: 100 μm (upper), and 20 μm (lower). The data are expressed as means ± SD (B, n = 3; D, n = 5).


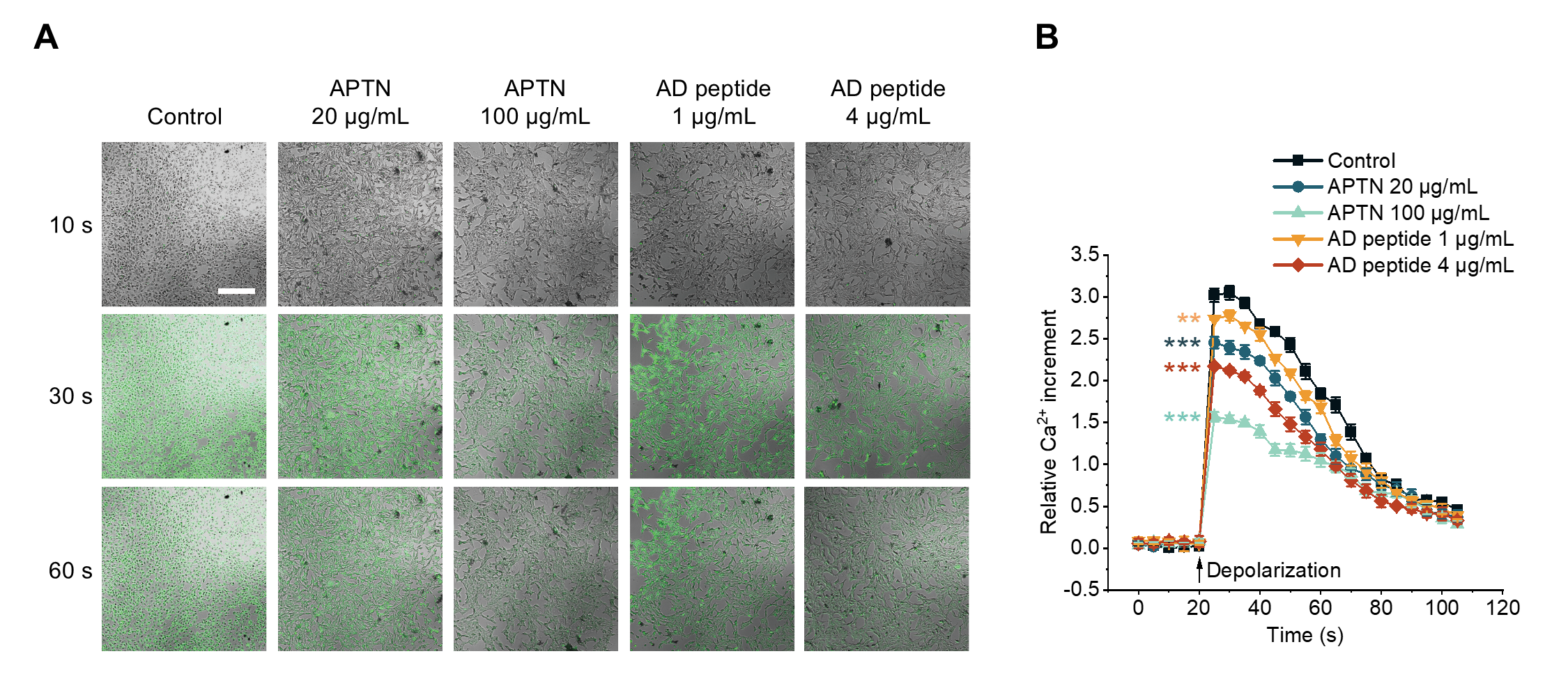


**Figure S13.** AD peptide and APTN inhibit calcium influx in depolarized neurons. (A-B) Representative fluorescence microscopy images (A) and quantitative data (B) showing the time course of intracellular calcium fluorescence intensity. Scale bar, 200 μm. The data are expressed as means ± SD (n = 5). **p < 0.01, ***p < 0.001, compared to the control group at the same time point.

**
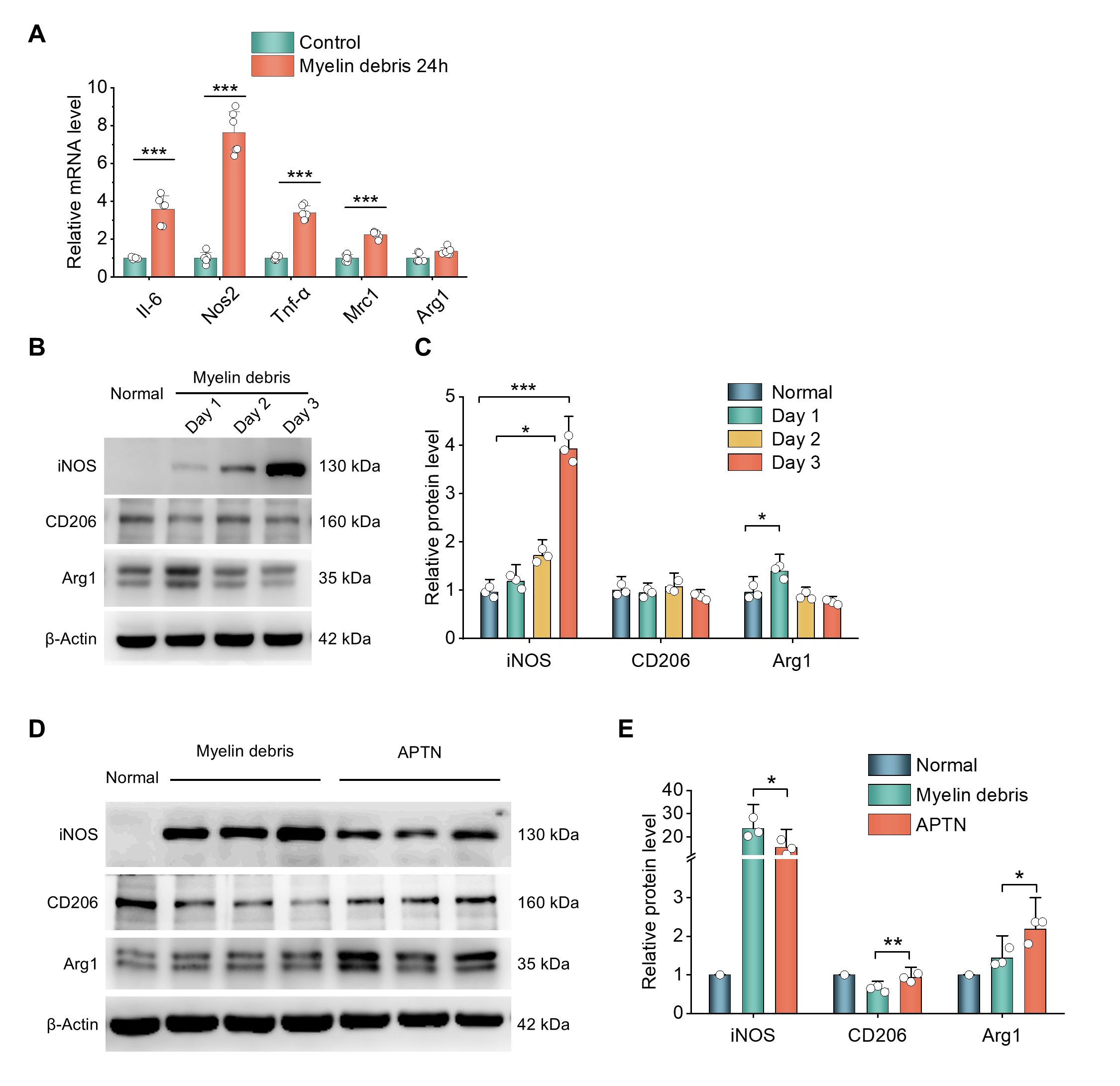
**

**Figure S14.** The effects of APTN treatment on the macrophage polarization induced by phagocytosis of myelin debris. (A) qPCR analysis of the mRNA expression levels of genes associated with the macrophage polarization in BMDMs incubated with myelin debris for 24 h (n = 6). (B-C) Typical Western blotting bands (B) and quantification data (C) showing the protein levels of Arg1, iNOS, and CD206 in BMDMs after incubation with myelin debris for 3 days (n = 3). (D-E) Typical Western blotting bands (D) and quantification data (E) showing the protein levels of Arg1, iNOS, and CD206 in BMDMs after phagocytosis of myelin debris and treatment with 50 μg/mL APTN (n = 3). The data are expressed as means ± SD. *p < 0.05, **p < 0.01, ***p < 0.001.


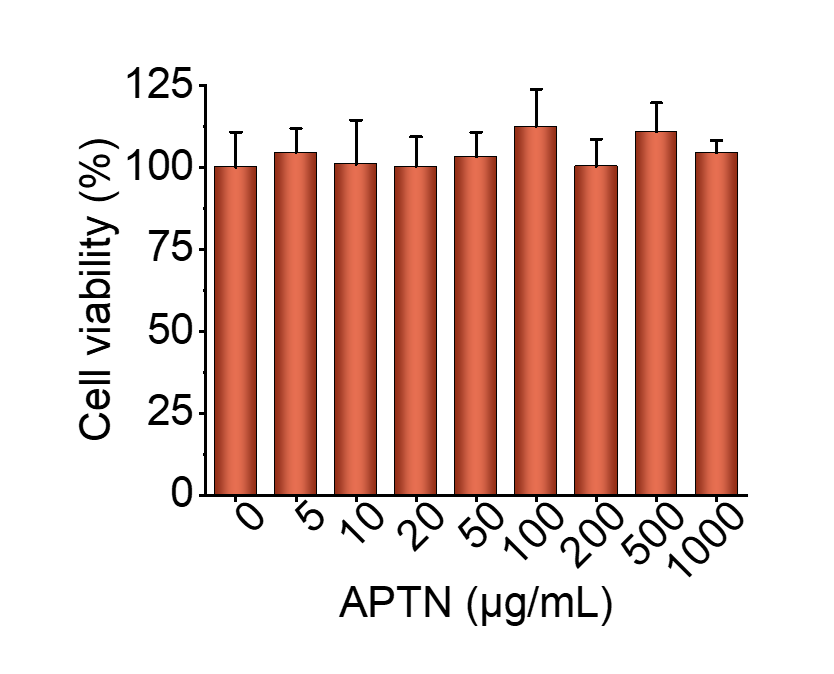


**Figure S15.** In vitro cytotoxicity evaluations of APTN in PC12 cells after incubation for 24 h. The data are mean ± SD (n = 3).


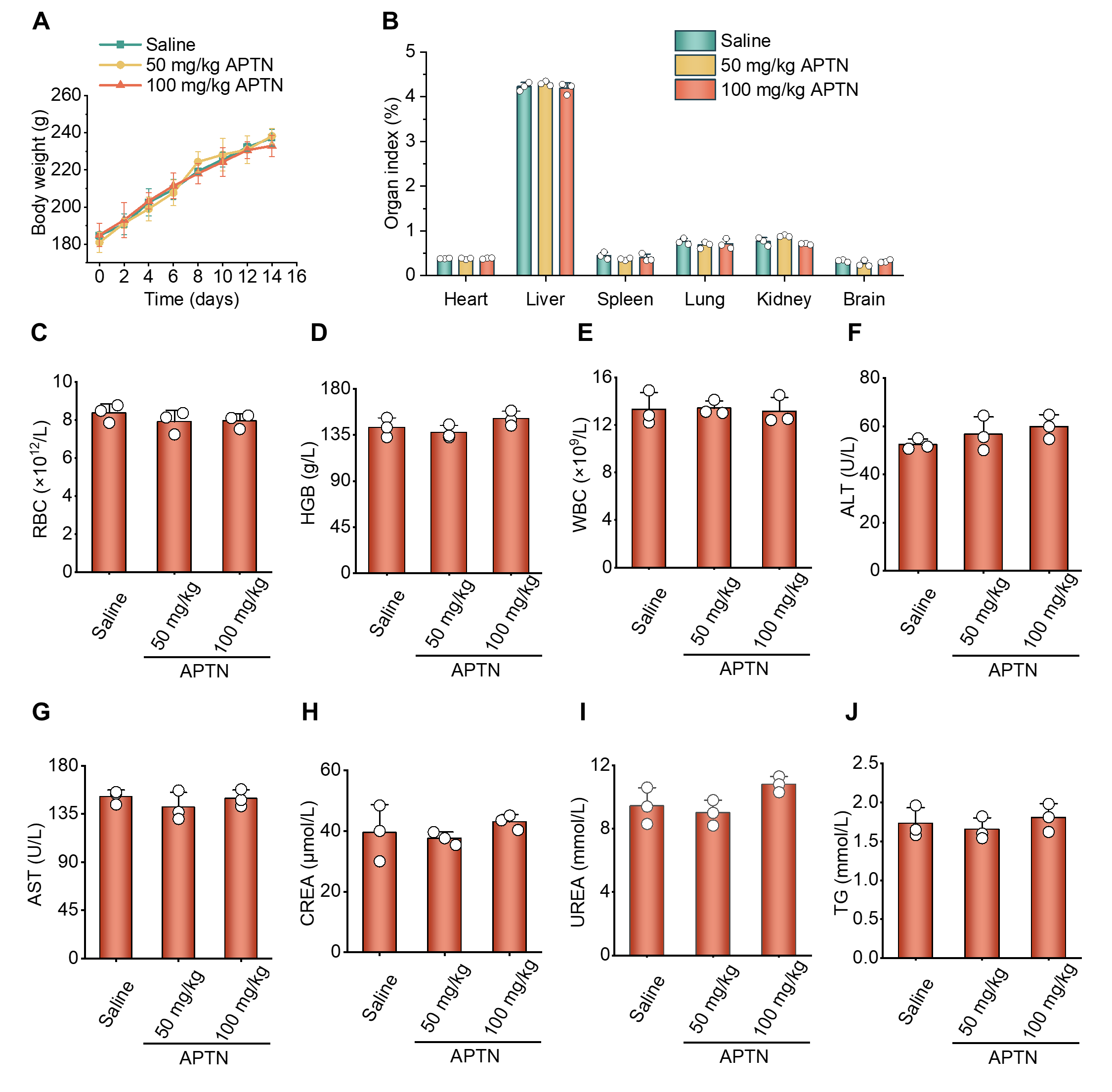


**Figure S16.** In vivo safety evaluation of APTN in rats. (A) Changes in the body weight of rats after a single i.v. injection of APTN at 50 or 100 mg/kg. (B) The organ index of the heart, liver, spleen, lung, kidney, and brain at day 14 after different treatments. (C-E) Typical hematological parameters of the peripheral blood for different groups, including RBC (C), HGB (D), and WBC (E). (F-J) Typical biochemical markers in the serum for different groups, including ALT (F), AST (G), CREA (H), UREA (I), and TG (J). RBC, red blood cells; HGB, hemoglobin; WBC, white blood cells; ALT, alanine aminotransferase; AST, aspartate aminotransferase; UREA, blood urea; CREA, creatinine; TG, triglyceride. Data are mean ± SD (n = 3).


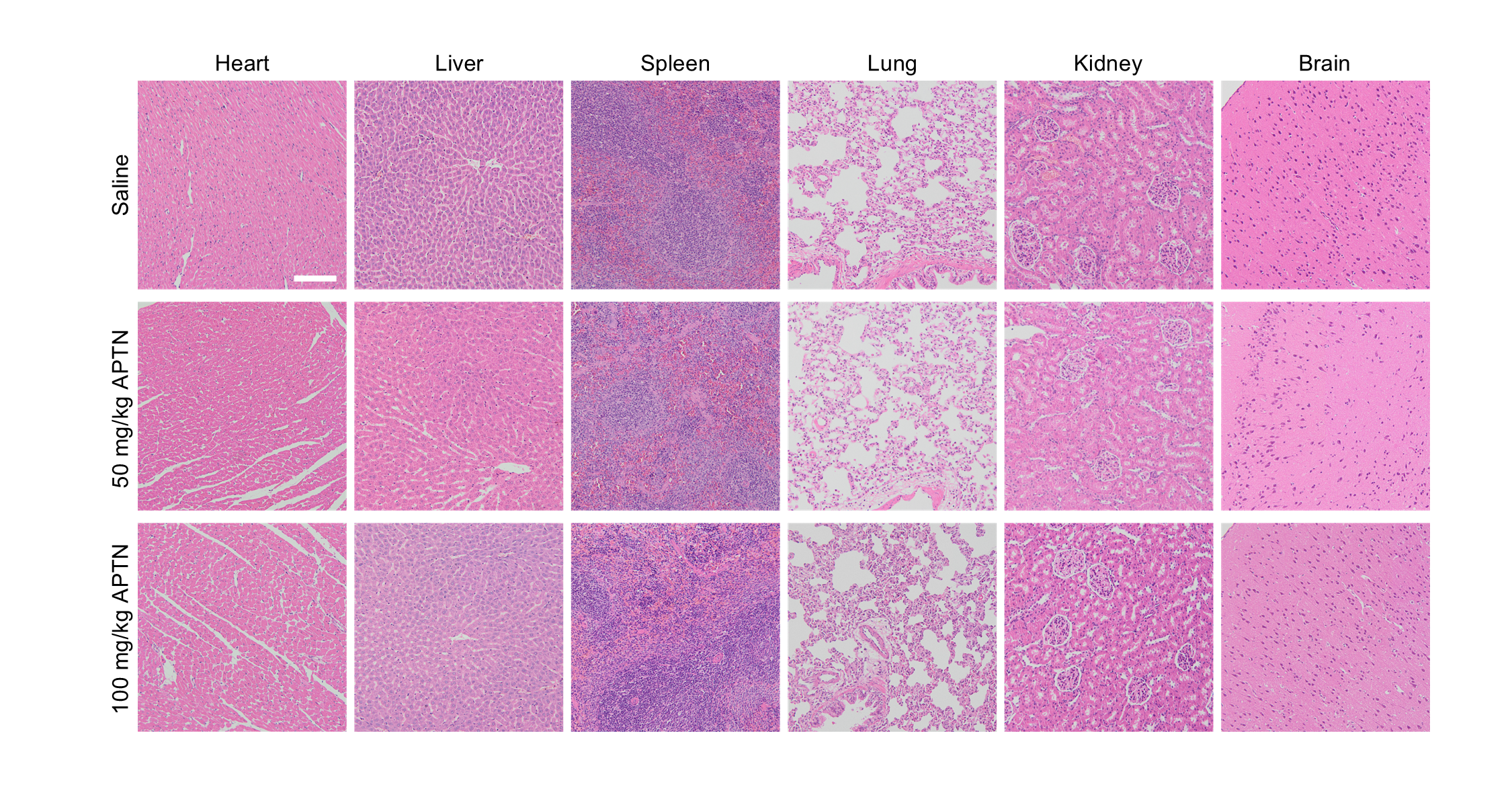


**Figure S17.** Histological evaluations of major organs of rats after a single i.v. injection of APTN at 50 or 100 mg/kg. The major organs including the heart, liver, spleen, lung, kidney, and brain were obtained from rats at day 14 after injection. Scale bar, 200 μm. The sections were observed by H&E staining (n = 3).
